# Advanced Formulation Approaches for Ocular Drug Delivery: State-Of-The-Art and Recent Patents

**DOI:** 10.3390/pharmaceutics11090460

**Published:** 2019-09-06

**Authors:** Eliana B. Souto, João Dias-Ferreira, Ana López-Machado, Miren Ettcheto, Amanda Cano, Antonio Camins Espuny, Marta Espina, Maria Luisa Garcia, Elena Sánchez-López

**Affiliations:** 1 Department of Pharmaceutical Technology, Faculty of Pharmacy, University of Coimbra, 3000-458 Coimbra, Portugal; 2 CEB—Centre of Biological Engineering, University of Minho, Campus de Gualtar 4710-057 Braga, Portugal; 3 Department of Pharmacy, Pharmaceutical Technology and Physical Chemistry, Faculty of Pharmacy, University of Barcelona, 08028 Barcelona, Spain; 4 Institute of Nanoscience and Nanotechnology (IN2UB), University of Barcelona, 08028 Barcelona, Spain; 5 Centro de Investigación Biomédica en Red de Enfermedades Neurodegenerativas (CIBERNED), University of Barcelona, 08028 Barcelona, Spain; 6 Department of Pharmacology, Toxicology and Therapeutic Chemistry, Faculty of Pharmacy and Food Sciences, University of Barcelona, 08028 Barcelona, Spain

**Keywords:** ocular drug delivery, patents, bioavailability, anterior eye segment, posterior eye segment, targeted drug delivery

## Abstract

The eye presents extensive perspectives and challenges for drug delivery, mainly because of the extraordinary capacity, intrinsic to this path, for drugs to permeate into the main circulatory system and also for the restrictions of the ocular barriers. Depending on the target segment of the eye, anterior or posterior, the specifications are different. The ocular route experienced in the last decades a lot of progresses related with the development of new drugs, improved formulations, specific-designed delivery and even new routes to administer a drug. Concomitantly, new categories of materials were developed and adapted to encapsulate drugs. With such advances, a multiplicity of parameters became possible to be optimized as the increase in bioavailability and decreased toxic effects of medicines. Also, the formulations were capable to easily adhere to specific tissues, increase the duration of the therapeutic effect and even target the delivery of the treatment. The ascending of new delivery systems for ocular targeting is a current focus, mainly because of the capacity to extend the normal time during which the drug exerts its therapeutic effect and, so, supplying the patients with a product which gives them fewer side effects, fewer number of applications and even more effective outcomes to their pathologies, surpassing the traditionally-used eye drops. Depending on the systems, some are capable of increasing the duration of the drug action as gels, emulsions, prodrugs, liposomes, and ocular inserts with hydrophilic properties, improving the absorption by the cornea. In parallel, other devices use as a strategy the capacity to sustain the release of the carried drugs by means of erodible and non-erodible matrices. This review discusses the different types of advanced formulations used for ocular delivery of therapeutics presenting the most recent patents according to the clinical applications.

## 1. Introduction

The ocular globe is an inimitable organic structure which comprises spectacular anatomic, histological, and physiological features. Mainly, the ocular globe may be segmented in two chief parts—the anterior and posterior portions. The anterior portion pertains about one-third of the eye and is constituted by the aqueous humor, conjunctiva, cornea, iris, ciliary body and lens, with the remaining second-third occupied with the posterior portion, made up of the choroid, neural retina, optic nerve, retinal pigment epithelium, sclera, and vitreous humor [1,2,3]. According with the segment of the eye elicited, several diseases can be pointed out. For the anterior segment of the eye a diversity of illnesses can be named as conjunctivitis, anterior uveitis, or cataracts. The posterior portion of the eye is affected by other pathologies such as age-related macular degeneration and diabetic retinopathy. Due to this, therapeutics of the eye constitutes a challenge as it must comprise the formulation of specific drugs with fine-tuned aspects to eliminate several problems related to barriers existent into the eye structure, such as precorneal tissues, and the side effects commonly observed in the available products in the market. Despite this, the most comfortable preparation for eye purposes are topical eyedrops, specifically to treat diseases of the anterior portion of the eye. Another drawback in the formulation aspect of drugs is related with the reduced time during which the formulation remains inside the ocular globe, determining the success (or not) of a therapeutic. The past decades saw research in the design and experimental analysis of new formulations aimed at increasing the residence time in the ocular tract as well as developing safe and cost-effective systems capable of increasing the permeation through different means [4,5]. To access the anterior segment of the eye, the commonly-used strategies are now concerned much more with enhancers for viscosity and permeation applied to solutions and to other formulations such as emulsions, ointments, and suspensions. In the last years, a significative number of nanosized formulations were introduced in the market for healing purposes of the anterior portion of the eye [6].

In another line, the posterior portion of the eye has been deeply studied to develop methods capable of safely delivering specific drugs to treat conditions related with chronic vitreoretinal illnesses. With these advances, further improvements are aimed to improve the capacity to penetrate the ocular barriers and to eliminate majority of the side effects verified with usual formulations [7,8]. Other factors are also essential, such as the ease of formulation, the reduction or even elimination of irritation in the ocular tissues, increasing the time of permanence in the precorneal tissues with improved bioavailability, and the capacity to sustain the delivery of the drugs. The current progressions in the research field of ocular systems do require the assiduity of scientists specialized in the formulation of drugs to establish a rationale through the assay of designing a system until the synthesis of this and consequent improvements in its features. The available review articles all point to the gradually increased presence of nanoparticulated systems for these purposes with other strategies also attained as the combination of traditional systems with novel techniques to improve their efficacy [7].

To surpass the boundaries imposed by the ocular globe to the delivery drug systems and, concomitantly, to enhance the bioavailability of the administered formulations, several novel systems were specifically designed to circumvent such problems. It is the case of aqueous gels, dendrimers, emulsions, implants, liposomes, nanomicelles, nanoparticles, ointments, suspensions, and many others forward described. The current review aims to make available a vast array of the patented available systems for therapeutics of ophthalmic illnesses [9].

## 2. Drug Delivery Systems for Ocular Route

Drop instillation performed by a topical path to deliver a given drug into the lower precorneal segment of the eye is a broadly known method to make an administration with the compliance of the patient. Nevertheless, from the original applied dose, only about 20% is kept in the precorneal segment of the eye as a consequence of the reflux phenomena happening during eye blinking. The diffusion of the drug across the corneal tissues is largely driven by the concentration of drug accessible in the precorneal sites. Yet, to achieve an effective ocular distribution of the drug using eye-drops it is mandatory to achieve a higher retention time in the corneal tissue and elevated corneal permeation [3,10].

Ocular drug transporters may alter drug’s efficacy and therefore, an evaluation of the interactions of new drug molecules and drug delivery systems with selected transporters is required during drug development [11]. These ocular transporters can be divided into two major families: the solute carrier (SLC) family and the ATP-binding cassette (ABC) family. SLC transporters use facilitated diffusion or couple an ion or electrochemical gradient to transfer their substrates across the cell membrane. ABC transporters use adenosine triphosphate (ATP) as the energy source [11].

Aiming the last feature some techniques as prodrugs, iontophoresis, cyclodextrins or agents capable to form ion-pair are considered. Nowadays the pharmaceutical markets present a wide offer on ophthalmic products and, in 70% of the cases, the prescribed medicines are mainly eye drops. This fact is supported by the easiness of application of this formulation, the compliance and suitability by the patients, the stability of the formulation and the relation cost-effectiveness [12,13,14]. However, there are a wide variety of different ophthalmic formulations (Table 1).

### 2.1. Conventional Topical Formulations

#### 2.1.1. Eye Drops

The formulations of eye drops possess a variety of factors that turn them into a widely-prescribed form (Table 2). They are safe, take immediate action after applied, have high patient-compliance, are suitable for the product, and, above all, are non-invasive. 

The application of eye drops is characterized by a permeation of the supported drug by a pulsatile mechanism which occurs subsequently to the topical instillation of the drops, with further quick declination of the drug concentration following a kinetic profile corresponding roughly to a first order elimination process. In consequence, to expand the time of contact of the drug, the capacity of permeation and the critical bioavailability of the desired molecule(s), several modifiers are powerful enough to be operated in eye drop formulations, such as cyclodextrins, enhancers of viscosity and of permeability [3,5,15,16]. 

With an enhancer of viscosity, it is possible to reach some variation in important variables such as the bioavailability and the time of permanence in the precorneal tissues. The most widely-used viscosity enhancers are hydroxyethyl cellulose, hydroxymethyl cellulose, sodium carboxymethyl cellulose, and a combination of hydroxypropyl methyl cellulose and a polyalcohol. The employment of these substances aims to augment the uptake by corneal tissues through a mechanism of slight disruption of the integrity of the mentioned tissue. Many other compounds are reported to possess similar properties in this context such as preservatives, chelating compounds, bile salts and surfactant molecules, being applied as permeation enhancers. The appliance of these enhancers in the context of ocular solution aims to considerably increase the bioavailability of the compounds; in the studies already performed just a few demonstrated the presence of toxicity associated with these substances. Still, the researchers are continuously evaluating the potential of toxicity of every new developed compound with the finality to assess its potential to be applied in the formulation of new products to improve the ocular therapeutics [31,32,33,34,35]. 

Researches carried out by Hornof et al. exposed the fact in which a compound used as an excipient, polycarbophil-cysteine, did not affect the tissue structure of the eye and was considered safe enough to be applied in the production of formulations for ocular route delivery [36]. The remarkable cyclodextrins are also considered agents capable of encapsulating hydrophobic compounds, in this case drugs, and promoting dissolution in hydrophilic substances as an aqueous solvent. The present detail is decisive for better delivering drugs into the surface of membranes of biological systems. Nevertheless, it must be considered that a cell membrane is constituted of lipophilic compounds, phospholipids, and cholesterol, mainly, which are not compatible for dissolution purposes with hydrophilic substances. On behalf of such, cyclodextrins do not pass through the membrane of the cell, but the encapsulated drug may do so by releasing from the inner core of the formulation and penetrating the biological barrier. For ocular targeting the best bioavailability of a given compound was achieved using cyclodextrins in concentrations lower to 15% [14,37,38].

Among the techniques used to get closer to an optimal bioavailability, excipients with diversified functions as cyclodextrins or enhancers of viscosity could be found. The relatively lipophilic membrane of the corneal tissue has low affinity for the hydrophilic cyclodextrin molecules and therefore they remain in the exterior aqueous membrane being cleared by the lacrimal fluid.

Regarding the molecules’ ability to act as penetration enhancers, their choice must be carried out according to their potential to sensitize the eye globe. According to these facts, the development of other compounds with improved properties and without harmful effects, regarding the targeted eye structures, has been explored in the last decades. As so, emulsions, ointments, and suspensions were explored attempting to achieve better features such as bioavailability of the encapsulated drugs, increased time of residence in the precorneal tissues, and higher solubility values [39,40,41]. Despite the current state-of-the-art and industry methodologies with regard to the manufacture of novel products based on nanotechnological approaches, the traditional formulations are still significantly marketed. Yet, the prior formulations have serious side effects associated to their employment, such as ocular tissues irritation, inflammation, inequal stability, and blurred vision. The recent research focusses mainly in improving the described critical features and reducing the side effects imputable to these formulations in order to produce better performances in an in vivo organic environment, avoiding serious undesirable effects. A lot of efforts are being made to try to carry the desired drugs to the posterior tissues of the eye using common formulations. In the next sections are described, for each formulation, the particular advances attained until the present and the overall perspectives for the next few years in what does respect to formulation advances. 

#### 2.1.2. Emulsions

The formulations with an emulsion nature are keen to increase the bioavailability of the encapsulated drugs as well as the solubility. The principal formulative mechanism to yield this design is based on two types of emulsions—oil-in-water (O/W) and water-in-oil (W/O). To be applied in the delivery of drugs to ocular tissues the favorite type is the O/W rather than W/O, mainly due to the favorable characteristics as diminished irritation induced in the target tissues as well as improved tolerance of the eye to O/W emulsions [5,42,43]. Some current examples of available medicines based on the technology of emulsion are the AzaSite^®^, Refresh Endura^®^ and Restasis^TM^ [44,45,46]. A vast array of scientific studies was able to validate the advantages related with the use of emulsions as the increased time of residence in precorneal tissues, the increased permeation of drugs in the corneal tissues, the capacity to keep the release of the drug and, so, rising the bioavailability of the drug [47,48].

Recently, a study conducted by Tajika et al. proved the increased anti-inflammatory capacity of a derivative of prednisolone, [3H]-difluprednate, at a concentration of 0.05%, formulated as an emulsion. The results carried out in the rabbit eye model proved that the designed emulsion was successful in delivering the therapeutic content only to anterior eye tissues with a very small amount of drug contacting with the posterior portions of the eye after single or multiple instillations. Once the single and several instillations of topical drops were applied, the tests to reveal the location of formulation through detection of radioactivity revealed, by decreasing order of intensity, the sites where the formulation was spotted: cornea, ciliary body of the iris, retina-choroid, conjunctiva, sclera, aqueous humor, lens, and vitreous humor. Overall, the quantity that entered the systemic circulation was insignificant. Subsequently to a period of 168 h, about 99.5% of the radioactivity was detected in both feces and urine. Based in the anterior evidences it can be firmly proposed that difluprednate emulsion may be considered as a probable product for the therapeutic of inflammatory ocular conditions [49].

Several emulsions fabricated using additives of lipidic sources as soybean lecithin or stearylamine were scrutinized to be carriers for drugs as azithromycin aiming to increase the parameters of bioavailability and retention in the corneal tissues. To assess the variation of the features of elimination pharmacokinetics for tears, three distinct doses of azithromycin—3.0 mg/mL, 5.0 mg/mL, and 10 mg/mL—in two different formulations types—emulsion and solution—were used. To accomplish so, the animal model of rabbits was used to perform the studies in vitro after a drop administration by topical route. In relation to the emulsion, it was perceived that the formulation was suitable as a delivery instrument and even braked the release of drug and, due to this, enhanced the chemical stability of azithromycin at two points—pH of 5.0 and 7.0—compared to usual aqueous solutions, and increased the time of permanence in the precorneal tissues. Combining the results, it is possible to perceive that emulsions with a strong lipidic character may be robust candidates for future products aiming the ocular delivery of drugs [50]. 

Another premise was stated and tried—the derivatization of active pharmaceutical ingredients with concomitant combination of the drug and an emulsion to improve the bioavailability in the ocular tract. This methodology offers the possibility of reducing irritation in the eye globe and enhancing the effect of the drug. To assess such idea the research team of Shen and collaborators tried to enhance the bioavailability character of a derivate of a commonly used drug—flurbiprofen acetyl—employing to achieve this finality a lipidic excipient, castor oil, and a stabilizer, Tween 80, to properly manage the formulation. The experimental design included four distinct formulations, varying both the concentrations of castor oil and Tween 80, between 0.1% to 2.5% and 0.08% to 4.0%, respectively. They found one of their four formulations suitable to massively increase the drug concentrations in the aqueous humor and the parallel studies of biocompatibility showed reduced capacity to induce ocular irritation [51]. 

Other studies carried out by other teams of researchers revealed that supplementary polymers to coat the emulsion formulations may also be appropriate as it is chitosan or an ether of hydroxypropyl methyl cellulose (HPMC). The posterior outcomes proved the efficacy of chitosan as coating in the improvement of the permanence time in the precorneal tissues with a consequent increase in the ocular bioavailability [52]. Another study established a comparison between an emulsion made of castor oil and polysorbate-80 plus a content of indomethacin, and a parallel formulation made with the same substances but with and additional coating with chitosan. The animal model used was the male albino rabbit and the method used to apply the formulation was the drop instillation by topical route. Results proved an increased time of residence in the tear fluid of the coated formulation in relation to the non-coated by a factor of 1.5-fold. Also, to complement the studies, the concentrations of the drug were assessed in the corneal tissues, conjunctiva and aqueous humor being 5.3 and 8.2-fold higher in cornea than in conjunctiva or aqueous humor, respectively [53]. Moreover, Muchtar and colleagues developed a submicron emulsion as ocular vehicle for delta-8-tetrahydrocannabinol, due to the low drug solubility, in order to decrease the effect on intraocular pressure in rabbits [54].

#### 2.1.3. Ointments

Also included in the category of topical formulations ointments are used too as carrier systems for drug delivery. The usual melting points of these systems are near to the ocular temperature of 34 °C due to their constitution base on a combination of solid and semisolid hydrocarbon molecules. But what hydrocarbon should we choose for a determined purpose? This simple detail produces much difference in the biocompatibility of a formulation as the substance must not be rejected by the body in order to prevent further organic side reactions and even to warrant the effectiveness of the purposes of the formulation to increase the bioavailability of the drug and to sustain the process of delivery [55,56,57,58].

The antibiotic vancomycin has in its backbone a structure of glycopeptides with proved outstanding activity against the effects of the aerobic and anaerobic microorganisms which stain positive by the Gram technique and the *Staphylococcus aureus*, resilient to the therapeutics with methicillin and cephem [59]. Despite this extraordinary therapeutic effect, few or even none of the topical formulations were available to be commercialized [60]. Improved outcomes related to the permeability of healthy ocular tissues to vancomycin were not projected. Despite this, slight positive results were observed in clinical trials for ocular pathologies, with this drug. But why such achievements? One of the reasons pointed out is related with the possibility of breaks in the barrier of the ocular globe leading to less selectivity in the uptake of foreign compounds with consequent elevated permeation [61]. 

Fukuda and collaborators wanted to study the dynamics of vancomycin hydrochloride formulated as ointments for ophthalmic purposes in the interior environment of the eye of rabbits linking this phenomenon with the extraocular infection with MRSA. To determine the minimum concentration which inhibits the development of the MRSA in bacterial infections, the investigators used a control group of rabbits, and as experimental condition a group infected with *Bacillus subtilis*, this last group generated using an intracorneal injection of a solution of *Bacillus subtilis* into the central part of the parenchyma. The value for the prior parameter ranged the 1.56 μg/g. After application of the ocular ointment formulation to both groups, with a concentration of 1.0%, it was verified that in the infected group the concentration was 3.68 μg/g ± 1.38 μg/g within 240 min after administration while in the normal group the values were of 0.49 μg/g ± 0.97 μg/g only after 120 min showing that *Bacillus subtilis* infected animals would benefit from a better therapeutic performance of the antibiotic molecules [62].

In an additional study, carried out by Eguchi et al., an array of four distinct ointment formulations was prepared through variation of the vancomycin concentrations—0.03%, 0.1%, 0.3%, and 1.0%—in a mixture of paraffin and Vaseline in a proportion of 1:4. To further assess the efficacy of the designed formulations the rabbit model with induced MRSA keratitis was used and the parameter evaluated after topical application of the formulation. The results demonstrated that the concentrations below 0.3% were not successful in avoiding the formation of subsequent lesions to the infection as abscesses. Nevertheless, the prior mentioned concentration was believed to be ideal for formulation as a therapeutic option for corneal keratitis derived from the MRSA infection as the results were keen enough to validate a period of 14 days without relapse of the infection [63].

Even though actual researchers push harder the limits of knowledge in this field to attain better efficacy on the developed materials, there is a parallel existence of other problems related with everyday formulations in use as emulsions, ointments, and suspensions, largely recognized by their side effects induced in the eye as blurred vision, redness, or itching. Another topic with possible implications is related with the continuous/chronic administration of the prescribed drugs, risk factor to achieve future systemic worries. To circumvent these actions and to improve the overall quantity of a given drug administered to the eye the exploratory strategies use modern approaches based on nanotechnological achievements because of the optimized properties of these last systems [64,65,66]. The following sections will present and discuss briefly the contribution of several formulation systems to the advances observed in the last decades in the field of pharmaceutics.

#### 2.1.4. Suspensions

These pharmaceutical forms are chemically defined as being dispersions of drugs, using for such purpose a hydrophilic solvent possessing an agent of dispersion or a suspension obtaining a final solution with a saturated character. Furthermore, this type of formulation will be suitable once it does not require an invasive application method. The particles which constitute the suspension are taken by the precorneal tissue, enhancing the contact time of the drug with the tissues, and also increasing the period during which the drug is therapeutically active. One of the particularities of these systems is the required well-designed size of the particles constituting the formulation, because this parameter affects directly the duration of the drug effect [67,68]. Using the TobraDex^®^ formulation it was verified in the rabbit animal model that the drugs dexamethasone and tobramycin exhibited elevated concentration values. Overall, the higher the size of the particle, the longer the retention time will be in the organism, and the slower the dissolution of the drug. On behalf of this it is anticipated that particles with an suitable size produce an excellent activity of the drug [69]. A couple of medicines are available worldwide to be employed in the treatment of infectious diseases. TobraDex^®^ is a suspension composed by a combination of an antibiotic (tobramycin at a 0.3% concentration) plus a steroid drug, dexamethasone (at a 0.1% concentration), aimed to treat patients with a positive feedback to the steroid therapeutics. This product had high viscosity, which was its foremost disadvantage the [70,71]. Fresh efforts leaded by Scoper et al. tried to diminish the values of the referred parameter for the prior product and to enhance its capacity to efficiently eliminate microorganisms. The aims of this project were to develop a novel suspension with improved features related with quality, kinetics, and permeation through tissues. Merging efforts lead to the development of TobraDex ST^®^, a new formulated suspension consisting in tobramycin at a concentration of 0.3% and dexamethasone, a steroidal molecule, at a concentration of 0.05%. The results demonstrated a reduced settling for TobraDex ST^®^ suspension in comparison to control, TobraDex^®^, with values of 3.0% for the first, over a period of 24 h. Also, the innovative formulation had higher effectiveness in the therapeutic effects against the action of *Pseudomonas aeruginosa* and *Staphylococcus aureus* and, in clinical trials with humans, the new formulation achieved augmented dexamethasone concentrations in the aqueous humor comparing to the TobraDex^®^ [69,72]. With this background is possible to conclude that the novel developed formulation, TobraDex ST^®^, has the potential to substitute the TobraDex^®^ marketed formulation due to four mainly characteristics—augmented bactericidal effect, improved pharmacokinetics, upgraded intrinsic features of the formulation, and enhanced compliance of the patients.

Another project to evaluate the performance of the drug rebamipine, as a suspension to treat the condition of dryness of the eye, was carried out through a multicenter clinical trial of phase II, double masked and randomized, conducted for a period of 4 weeks. The conditions applied were a placebo as control and two different concentrations for rebamipide, 1.0% and 2.0%. The most important parameters—efficacy and safety of the formulation—were determined using humans after a topical instillation procedure. Obtained results incorporated several different characteristics. Using the staining methods of Lissamine green for conjunctive membrane and fluorescein for cornea tissues at determined periods of 2 and 4 weeks it could be inferred that the outcome were dose-dependent in the three tested conditions: Placebo, 1.0%, and 2.0% concentrations. Respective to the production of tears it was observed no significative statistical alteration, in comparison to the control, between the first day to the fourth week. Despite such, the produced film of tears was significantly altered in the assays using the concentrations of 1.0% and 2.0% of rebamipide, namely in the break-up time parameter when compared to the placebo. Also, comparatively to placebo, the patients reported enhancements in their conditions. The occurrence of adverse effects was also analyzed with dysgeusia, nasopharyngitis, and irritation of the eye globe increasingly reporting from the placebo, passing through the 1.0% to 2.0% concentrations of the drug. In spite of these occurrences the side effects disappeared after retrieval of treatment and the studies proved that the formulated suspensions are tolerated and present effectiveness in the treatment of dry eyes conditions. Weighting the outcomes of the two different concentrations allowed the realization that the 2.0% concentration was more effective than 1.0% formulation [73].

### 2.2. Nanotechnological Inspired Delivery Systems

The last decades of scientific progress were extremely fructiferous regarding therapeutic developments for ocular diseases treatments. The uprising of nanotechnology in combination with already well-established pharmacological compounds lead to the currently used tactics in the treatment of both anterior and posterior portions of the eye (Table 3). 

**Figure 1:**
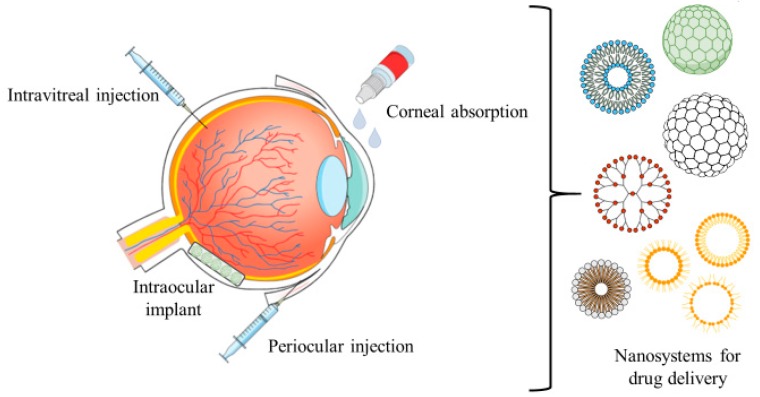
Ocular routes for drug delivery systems.

With the proper fine-tuning, the nanoparticulated systems may assure reduced side effects with augmented bioavailability and better capacity of absorption. Several examples are used in nowadays practice being nanocarriers: Dendrimers, liposomes, nanoparticles, nanosuspensions, and nanomicelles are just a few cases of the available arsenal for ocular treatment of pathological issues with many of these materials demonstrating auspicious outcomes (Figure 1) [74,75].

#### 2.2.1. Nano-Enhanced Contact Lens

Contact lenses cover the cornea using their curved shape and thin surface. Once applied they adhere to the wet surface in the exterior of the eye mainly due to surface tension phenomena. A vast range of drugs have been formulated to be delivered through the ocular route such as antihistamines, antimicrobials, and β-blockers. The existence of the contact lens allows the drug molecules to stay for longer periods in contact with the ocular tissue with increased flux crossing the cornea and less of the drug lost to the lacrimal duct [91,92]. The common method to load the contact lens with a drug supply is to immerse them in a solution of a given drug. In performed studies the immersed contact lens demonstrated a better profile of release of drug in comparison to usual eye drops systems [93]. A study completed with dexamethasone demonstrated that this drug had higher bioavailability when formulated in a contact lens made up of poly(hydroxyethyl methacrylate) in contrast with eye drops [94]. Despite these advantages, the loaded lens has some drawbacks such as insufficient and irregular drug loading with further shorter time for release of the contents [71]. To outdo these limitations, two types of formulations were developed consisting of a contact lens loaded with drug-loaded nanoparticles and a contact lens printed on a molecular level. The first technique makes use of the known nanosystems such as liposomes or micelles to capture the drug and then disperse the systems in the material which constitutes the contact lens. Another study conceived contact lens loaded with nanoparticles to deliver lidocaine. These contact lenses were arranged through dispersion of drops of microemulsion containing lidocaine or liposomes in hydrogels made up with poly-2-hydroxyethyl methacrylate (p-HEMA). The results showed that lidocaine was released through a period of, approximately, 8 days, and contact lenses loaded with nanoparticulated systems were very auspicious for extended ocular delivery [95]. 

The contextual application of systems with stimuli-responsive features is a comprehensive tool to circumvent the previous referred problems [95,96]. Using variables such as temperature or pH the engineered particles may undergo reactions leading to the release of their content only in the ocular milieu, avoiding the prior issues. Regarding the manufacture and performance of contact lenses these were able to show good profiles of both encapsulation and release with regard to their drug content [95]. An experimental study using contact lenses compared the method of production with the lenses derived from molecular printing revealing a 1.6-times higher capacity to encapsulate the drug timolol in comparison to contact lenses fabricated using usual approaches. Also, the first lenses were able to endure the release of timolol [97]. An extra study using printed lenses containing the drug ketotifen fumarate (directly stamped) revealed a higher bioavailability of the drug in comparison to the usual process of soaking lenses or even to commonly-used eye drops. The relation between the parameter of bioavailability between the drug-stamped lenses and the non-printed lenses was 3-fold higher for the first referred [98]. Such results clearly demonstrate the capacity of the drug-stamped lenses to be much more effective in the delivery of drug to the desired tissue in relation to the other two referred formulations.

#### 2.2.2. Dendrimers

Dendrimers possess a star shape with a nanosized, multiple branched polymeric web. Dendrimers are accessible with several modifications regarding the terminals of the chains with multiple functional groups being present as amine, carboxyl, or hydroxyl groups. They also have a range and diversity of molecular weights. With the capacity of wear on dynamic chemical groups also comes the possibility of conjugation with other molecules selective according to the desired milieu. These systems require an optimization of their chief characteristics as functional groups, molecular geometry, charge of surface, and molecular weight, to obtain a system affordable to apply in the context of drug carrying. On behalf of the extraordinary structure of dendrimers, lipophilic or hydrophilic drugs may be encapsulated [99,100,101]. 

Regarding the ocular route purpose, the dendrimers using poly(amidoamine) (PAMAM) as structural basis are widely applied. In a study carried out by Vandamme and collaborators they stressed the possibilities in the application, as carriers, for ophthalmic miotic and mydriatic effects of the drugs, respectively, pilocarpine nitrate and tropicamide designed as PAMAM dendrimers [102]. 

To reach acceptable results the researchers used the variable residence time in the rabbit eye for fluorescein in saline formulation and fluorescein in PAMAM formulation. The bioadhesive control formulation applied was a 0.2% (*w/v*) Carbopol solution. The results demonstrated that both PAMAM and Carbopol solutions had considerably higher values for the residence time in relation to saline formulation. Because of this, the employment of dendrimers shall be an available opportunity to expand the previous referred variable in this study with further better therapeutic outcomes because of the increased bioavailability of the drugs. In parallel, the results also demonstrated that PAMAM formulation was keen to reveal increased miotic and mydriatic effects in albino rabbits.

Also, to prevent the formation of wound tissues because of surgery for filtration of glaucoma, immunomodulation, and blockade of angiogenesis were attained, respectively, through the application of novel synthesized compounds derived of dendrimers from PAMAM containing glucosamine (GA) and glucosamine 6-sulphate (GAS) with consequent diminution on the formation of scar tissue. The development of this study demonstrated a positive influence on the evading of formation of scar tissue after the referred surgery with the administration of GA and GAS [102,103].

#### 2.2.3. Intraocular Implants

These materials are pharmaceutically engineered to deliver the drug cocktail content in a determined location during a specified time range preventing the prior requirement of several injections in the ocular tissues with further developing inflammatory processes and infections. If aimed to deliver the drug into the posterior portions of the ocular globe these implants are physically settled in the proper place through a surgical procedure that requires a small incision in the region of the pars plana, being anteriorly located to the retina but posteriorly to the lens [104,105]. Despite the invasiveness of the method, it has achieved an increasing interest as a result of the positive returns when regarding to the capacity to relay during much longer times, releasing the same amount of drug to the tissues, evading the blood–retina barrier and diminishing the side effects related with the presence of the drug [106,107]. A variety of devices were designed and manufactured to be applied as ocular implants for therapeutic of illnesses with a chronic vitreoretinal nature. These devices are also obtainable in two different chemical structures—the biodegradable and the non-biodegradable ones. The latter are configured with zero-order kinetics, which means they can release for longer times. They use a chemistry based on various polymers to become available as ethylene vinyl acetate (EVA), polyvinyl alcohol (PVA), or even polysulfone capillary fiber (PCF) [108,109]. As actual examples of products already developed for this aim are Retisert^®^ and Vitrasert^®^. The first was approved by the Food and Drug Administration (FDA) as a pharmacological tool for the pathology of chronic uveitis on the posterior portion of the ocular globe. It consists on several foil of silicone with PVA. It allows a controlled release of fluocinolone acetonide for a period up to 3 years, which also proves that the implant is successful in controlling the inflammation with consequent better visual acuity and diminished reappearances of uveitis. Nevertheless, some side effects are present, like the possible development of cataracts and high intraocular pressure [110,111,112]. 

The second is an implant for intraocular delivery of ganciclovir using a controlled-release mechanism, also approved by the FDA, to treat cytomegalovirus retinitis caused by acquired immune deficiency syndrome (AIDS). The device possesses weighs 4.5 mg and has a shell made of PVA/EVA which allows the unhurried release of the therapeutic principle for a period ranging from 5 to 8 months. In parallel with this reality the product does not present systemic toxicity and is available at a reduced price [113,114,115,116]. 

The prior examples of devices reveal the potential of nanosystems in future therapeutics, but the non-biodegradable materials have the drawback of requiring a double surgery procedure—to implant and to remove the drug-releasing system—leading to a much more extensive treatment, possibly more painful for the patient and with less compliance. Additionally, other possible physiological occurrences may border the application of these inventions, such as cataracts, retinal detachment, or hemorrhage [117,118].

The intraocular implants are also available in the category of biodegradable products, which are receiving a considerable amount of attention by the medical public due to their biocompatibility and capacity to sustain the delivery of drugs. Biodegradable implants, including nanoparticles, are manufactured using polymers such as polylactic acid (PLA), PLGA, polycaprolactones, or polyglycolic acid (PGA), producing an instrument that does not require removal after implantation, a significant advantage in relation to others implants of non-biodegradable origin [118,119]. Some practical examples of medical devices used to this purpose are the biodegradable products for ocular deliver Surodex™ and Ozurdex^®^. The first is intended to treat macular edema whilst the second is purposed for the treatment of intraocular inflammation, both using dexamethasone as a therapeutic agent. Surodex™ was engineered with a lattice composed by hydroxypropyl methyl cellulose (HPMC) and PLGA which act as a polymeric core. It is further implanted in the anterior chamber of the eye to control the processes of inflammation after surgeries in patients with cataracts. The product can sustain the release of dexamethasone for a period between 7 to 10 days being the anti-inflammatory provided effect superior in relation to other topical corticosteroids [70,120,121]. 

Ozurdex^®^ was approved by June 2009 aiming to treat macular edema. It is an implant settled by intravitreal procedure with good features such as biocompatibility and biodegradability. This product also has a singular characteristic—it uses the technology NOVADUR^®^ to achieve a suitable delivery of dexamethasone. This technology uses the capacity of PLGA polymeric lattice to encapsulate the drug, and to slowly degrade and form monomers of glycolic acid and lactic acid, which allows for an extended release period up to 6 months. The submission of Ozurdex^®^ to accurate clinical trials emphasized the capacity of this product to reduce the overall loss of vision (potency) and upgrade outcomes with respect to vision perception in eyes possessing macular edema in association with branch or central retina vein obstruction. Ozurdex^®^ is also pointed, as a consequence of several clinical studies, to treat diabetic retinopathy and Irvine–Gass syndrome [122,123,124]. 

#### 2.2.4. In Situ Gelling Nanosystems

The designation used refers to the solution with a polymeric nature that may experience a transition of phases between solution and gel forming then a gel presenting viscoelastic features as a result of a stimuli from the surroundings. The process of gelation is also prompted by other modifications such as pH variations, temperature, presence or absence of ions, and incidence by ultraviolet (UV) radiation. To develop and apply a system such as this in the ocular structure the chief factor chosen was the temperature to further develop thermosensitive gels [125,126]. A vast array of polymers, sensible to variations in temperature and acceptable to be applied in the eyes, is described in the available literature. Among these compounds are molecules such as polyethylene glycol, poly(lactide), and poly(glycolide) and their derivatives, polycaprolactone or even chitosan (the latter being a highly positive polymer). These chemicals use a mechanism based on the formation of micellar aggregates when the temperature reaches a certain threshold, jellifying due to phenomena of packing or aggregation. Intended to be used as drug delivery vehicles these systems can be modeled to jellify after mixed with the desired drugs, both as solutions, which will further form a gel in the milieu of administration at a temperature between the physiological values [125,127,128,129]. Related to the anterior and posterior sections of the eye, these thermosensitive formulations revealed good results by increasing the bioavailability of the drugs [130,131]. As well, Gao and collaborators made a screening on the capacity of a gel with thermosensitive properties, constituted by a polymeric network with a frame A-B-A, PLGA-PEG-PLGA, to efficiently carry dexamethasone acetate into the ocular globe. The formulation was projected for a concentration of 0.1% (*w/v*) in 20% of the polymeric mixture previously described and then injected into a rabbit’s eye. In comparison to an eye drop formulation the concentration of therapeutic agent in the anterior segment of the eye was significantly higher in the polymeric formulation possessing also the highest area under curve (AUC) values. The ratio of increase in the polymeric formulation comparatively to the eye drop formulation was about 7.0 and 7.98 for the maximum concentration and AUC, respectively [132]. The outcomes from this study reveal the potential of a polymeric formulation to be used in the context of ocular delivery due to the increased bioavailability. Rieke et al. described the capacity to sustain the delivery of ovalbumin to the choroid and retina portion of the eye of a rat subsequently to the administration in the subconjunctival layer of the eye as a product—ReGel™—which, by turn, consists in a biodegradable and thermosensitive polymeric network of PLGA-PEG-PLGA with the previously-described molecule. The team assessed a period of 14 days during which they stated that in the choroid, retina, and sclera of the rats the concentration of ovalbumin was kept at detectable levels. A combination of poly(*N*–isopropylacrylamide) and poly(ethylene glycol) diacrylate as hydrogels was tested to attain a sustained release of macromolecules like immunoglobulin G (IgG) or bovine serum albumin (BSA). The results demonstrate that BSA was able to be prolongedly released for in vitro for almost 3 weeks [133]. In a recent patent, the inventors have designed polyethyleneglycol (PEG)-polyacetal (PA) copolymer and polyethyleneglycol (PEG)-polyacetal (PA)-polyorthoester (POE) copolymer for the drug delivery purposes. This solution form will convert to gel form at body temperature [134].

The studies previously described show a great capacity of these thermosensitive gels to reduce side effects of the employed therapeutic molecules, diminish the required posology, increase the bioavailability of drugs with parallel use of sustained release systems, and even extended contact time of the drugs with the cornea tissue. Overall, these thermosensitive systems constitute a suitable strategy to be employed for the delivery of drugs for ocular diseases [132].

### 2.3. Liposomes

These systems are composed by vesicles of lipid nature arranged on a single or multiple phospholipid bilayer design with a hydrophilic core. Liposomal systems are classified based on their size and on their bilayers of phospholipids. As so, they can be categorized as small unilamellar, with sizes between 10 nm and 100 nm, large unilamellar, with a range of sizes between 100 nm and 300 nm, and multilamellar if they have more than one bilayer and, so, are bigger than 300 nm [134,135]. Regarding the ocular application route, liposomes are here described as suitable systems to deliver drug content because of their high efficiency to encapsulate both hydrophilic and lipophilic drugs, high biocompatibility, and a structure similar to the natural cell membrane. Liposome formulations were accurate enough to efficiently delivery their contents in the anterior and posterior portions of the eye, according to numerous scientific studies [136,137]. A fresh study, developed by Natarajan and collaborators, on the delivery of latanoprost encapsulated within liposomes and applied in the anterior portion of the ocular globe demonstrated a capacity to reduce the intraocular pressure in the eye of a rabbit, for a time range of 50 days [138]. Related with the anterior segment of the eye, the actual frontline in research of the topic is pulling the limit on the residence time of drugs with precorneal tissues through the integration of lipids with positive charge or even polymers with mucoadhesive properties in the liposomes. Experimentally, the liposomes with cationic nature displayed improved capacity to deliver their content in the ocular tissues in comparison to negative or neutral charged liposomes. This phenomenon is a consequence of the interaction with the negative charge of the corneal tissues [139,140].

Liposomes with anionic and cationic shielding were arranged with an acyclovir content. To accomplish so dicetylphosphate and stearylamine were charged to play, correspondingly, the role of anionic and cationic inducers. After topical application into the eyes of the rabbits, the concentrations of acyclovir were assessed after 2.5 h exhibited the cationic liposomes in a superior concentration in the cornea tissues than anionic liposomes or acyclovir alone. The cationic liposomes were even more easily absorbed throughout the cornea tissues. Overall, these results point to the negative-charge nature of the surface of the cornea and to the best binding capacity of the positively charged formulations that interact electrostatically with the ocular structure being more easily absorbed as well as kept [141].

Another study demonstrated that the delivery of Coenzyme Q10 (CoQ10) in liposomes coated using trimethyl chitosan mucoadhesion strategies increased the residence time in the tissues of the precorneal segment of the eye of rabbit 4.8-fold [142]. Regarding other strategies for delaying the clearance of liposomes from eye tissues, as in the posterior portion of the eye, the hindering of enzymatic reactions or purely chemical degradation of unstable molecules as nucleotides of peptides is a desired aspect. As an example, it can be referred that fluconazole had its half-life intensified from 3.08 h to 23.4 h when administered as a liposome formulation in the vitreal portion of the eye [143]. In the same line of illustrative act, a study with tacrolimus formulated in liposomes to treat the condition of uveoretinitis, applied the product only once through an intravitreous procedure further observing that a concentration of 50 ng/mL was kept for a period of 14 days. The formulation was also revealed to decrease the intrinsic toxicity of the drug in the internal retinal cells and blocked the uveoretinitis more efficiently in contrast to the drug alone [144].

Currently, a vast array of pharmaceutical formulations using liposome technology are in survey with a restricted number being submitted to pre-clinical and clinical trials and an even scarcer number available commercially [145]. The products Tears again^®^ and Visudyne^®^ are clear illustrations of these formulations already available on the market to treat ophthalmic diseases. The first product consists in a spray made of liposomes of phospholipid nature for the conditions of dryness of the eyes which has successfully demonstrated clinical noteworthy returns in comparison to isotonic saline solutions and gels with triglycerides [146,147]. The second product is applied in the condition of macular degeneration age-related as a therapeutic to revascularize the subfoveal choroidal tissues using the liposomal formulated verteporfin, a photosensitizer employed in photodynamic therapy. Other illnesses on which it may be applied are myopia or histoplasmosis of the eye [148,149].

#### 2.3.1. Nanomicelles

Nanomicelles possess exterior hydrophilic polar heads and an interior hydrophobic fatty acyl chain which makes them capable of delivering poorly water-soluble drugs and of protecting molecules such as proteins or peptides. Such structures are amphiphilic and can possess in their nature an intrinsic capacity to be polymers or surfactants [150,151]. Fresh review studies performed by Cholkar et al. demonstrated the critical boundaries in the application of these systems for attaining the purpose of delivering drugs for the treatment of ocular tissues. The main advantages of such type of formulation are related with the ability to be easily prepared, the final small size they yield, and the tremendous capacity to encapsulate high quantities of drugs. As a direct consequence, the formulations carrying such morphology allow increased bioavailability of the drugs to be administered with further acquired improved clinical results [152,153]. Several clinical studies carried out with different drugs used the formulation format of nanomicelles. A study conducted by Civiale et al. settled nanomicelles fulfilled with dexamethasone using an objective copolymer of poly hydroxyethyl aspartamide (PHEAC) and its respective pegylated molecule to vector the formulation to the anterior portion of the eye globe of rabbits. To assess the concentrations of the drug, the aqueous humor was extracted and analyzed revealing that dexamethasone encapsulated in nanomicelles of PHEAC had increased values for bioavailability compared to usual suspensions of this drug with an overall quantity value 40% higher than the control suspension formulation. The results pointed to the worthwhile opportunity of applying these formulations of nanomicelles to deliver small molecules to the ocular tissue by topical application [154]. The nanomicellar systems were also used in gene therapy for the ocular globe. Another study, this time carried out by Liaw and collaborators, aimed to delivery certain genes into the corneal tissues using topical administration. A copolymeric lattice based on poly(ethylene oxide)-poly(propylene oxide)-poly(ethylene oxide) was reported to be a carrier for the delivery of selected genes incorporated into plasmids containing the DNA of interest fused with a reporter gene, in this case the *LacZ* transfected into mouse and rabbit eye tissues. After achieving the results, it was clear that these systems were a considerable possibility to transfer genetic information. Further researches with other genomic information—tissue-specific promoters—were conducted with the promoters keratocan and keratin 12. The overall expression of the genes of interest was assessed using the activity of another reporter gene which is translated into the functional β-Gal enzyme which converts a reagent into a color-identifiable molecule. The identifiable color was visualized after six days of continuous administration, three times a day, of pK12-Lac Z-PM, an eye drop formulation, applied in the corneal tissues of mice and rabbits. Also, the most feasible mechanism responsible for the process of transfection was the endocytic pathway combined with the relative size of the obtained particles through paracellular transference [155]. One of the most recent ocular products using nanomicelles is CEQUA™. This product is a cyclosporine ophthalmic solution at 0.09% is a calcineurin inhibitor immunosuppressant indicated to increase tear production in patients with keratoconjunctivitis sicca (dry eye) [156].

Other strategies being put in use are concerned with the use of nanomicellar systems to deliver the desired therapeutics to the posterior portion of the eye, a fact recently achieved. Aiming the cumulative validation of the previous obtained results other studies were performed but, instead, with voclosporin encapsulated in nanomicelles, in rabbit animal models. The outcomes demonstrated a good capacity to penetrate eye barriers and a parallel outstanding profile of delivery to the posterior region of the eye [150].

A research group governed by Ideta tried to distribute a fluorescent-conjugated compound, poly-l-lysine labeled with fluorescein isothiocyanate (FITC-P(Lys)) to the posterior portion of the ocular globe of the animal model trying to stimulate the angiogenesis. For such a purpose they used an intravenous injection of the drug formulation. The results showed that the administration of FITC-P(Lys) without previously formulating it resulted in the death of the animals 1-h post administration. On the other hand, the administration of FITC-P(Lys) previously formulated in a micelle network made of polyethylene glycol and poly-α, β-aspartic acid in a repetitive mesh lead to no animal death. Such outcomes reveal the presence of a non-existent quantity of drug in the formulated design. Pharmacokinetic assay revealed a peak of micellar concentration (maximum concentration), 4 h after a unique administration, in the portion of retina and choroid membranes. Additionally, the drug was also sensed 7 days after the administration. The perceptible longer times of circulation of the formulation are due mainly to the ratio between polymer and drug. The authors also stated that the extended times of circulation of micelles may increase the permeation and retention at the places where the angiogenic effects were required [157]. The great observed advantage is related to the selective accumulation of the formulation in ill tissues in comparison to normal tissues.

The presently-used strategies are devoted to deliver active pharmaceutical ingredients through a non-invasive route to both anterior and posterior portions of the eye. Following this principle, the actual trends in nanomicelles’ development are achieving more attention from experts due to the topical application strategy avoiding invasiveness. Using personalized skills such as the very small size attached to the presence of a halo of hydrophilicity nanomicelles may be held in the systemic circulation for extended periods, with an increased accumulation in the desired ocular portion, derived from the electrostatic interactions (negative–positive charged surfaces) and from the increased permeability of the damaged portions. Consequently, it allows the drug to be permeated and accumulated specifically in the ill regions. Yet, the design and engineering of the formulation regarding the choice of the polymers and surfactants employed is also a determinant step in the accomplishment of a final optimized product [158,159].

#### 2.3.2. Nanoparticles

The nanoparticulate systems are chemically characterized as colloidal transporters ranging from 10 nm to 1000 nm. In order to be applied in the ophthalmic tissues these arrangements required specific compositions to assure safety and efficacy: Proteins and lipids were employed as well as polymers from synthetic or natural sources, amongst them chitosan, albumin, sodium alginate, polylactic acid (PLA), poly(lactide-*co*-glycolide) (PLGA) or polycaprolactone. Also, the nanoparticle-encapsulating drugs may be divided in two principal devices: Nanocapsules and nanospheres. The first have the drug encapsulated in the interior of the polymeric lattice formed. The second have the drug homogeneously dispersed along the polymeric lattice. In recent years, nanoparticulated engineered forms apprehended the interest of several research groups to develop delivery systems intended to specifically carry drugs to anterior and posterior ocular tissues [160,161]. 

The small size of nanoparticles is a very good feature for their promising characteristics for diminished irritation in the corneal tissue and capacity to sustain the delivery of the drug with further avoidance of multiple administrations, which are related to therapeutic compliance. Nevertheless, these hydrophilic formulations may also be subjected to quick elimination from the precorneal compartment. On behalf of this, nanoparticles possessing mucoadhesive characteristics were prepared aiming to be topically administered, with consequent improved time of residence in the precorneal compartment. To enhance the time of permanence of the nanoparticles in the previous mentioned compartment some compounds are currently used as chitosan, hyaluronic acid, or polyethylene glycol (PEG) [162]. Among these, coating with chitosan is the preferred method to achieve the purpose referred to. As this molecule has a positive charge it is attracted by the negative surface of the cornea further improving residence time and diminishing clearance of the formulation [6,7,163,164]. In a study conducted with natamycin-encapsulated nanoparticles structurally made up of a mixture of chitosan and lecithin, it was proved that those particles were demonstrated high bioavailability in a rabbit ocular globe with both lower dosing and doses in comparison to the marketed suspensions reported for the same effect [165]. Another study carried out by Musumeci et al. stated that nanoparticles constituted with a mixture of PLGA-PEG and being further fulfilled with melatonin were successful in lowering the intraocular pressure in rabbits, comparative to nanoparticles engineered only with PLGA and further fulfilled with melatonin or even with other aqueous solutions. A proposal to explain the results is that nanoparticles manufactured with PLGA-PEG achieved a reduced zeta potential relative to PLGA-nanoparticles. Due to this, the first ones were successfully able to interact with the eye surface yielding a longer hypotensive effect [166]. 

As an alternative to the process of delivering of drugs to the posterior section of the eye, the nanoparticulate system aims to sustain the delivery for long periods. The main features which influence the penetration of nanoparticles into this portion are the size and the properties of the surface [167,168]. A periocular injection into the animal model ocular tissues, probably due to specific removal by such tissues as eye conjunctive and episcleral. Among the results it was verified that particles with a size ranging between 200 nm and 2000 nm were capable of being taken efficiently and kept by the tissues where they have been administered for a period up to two months; also, the nanoparticles with a small size, chiefly as a consequence of their capacity to rapidly release the content and be cleared, are not capable of sustaining the drug levels at the retina [169]. After the application of the intravitreal injection the nanoparticulated systems travel via several layers of the retina. The capacity of the nanoparticles to pass from the vitreous humor to various retinal tissues is mainly due to the properties of the nanoparticles’ surface. 

With the previous mentioned factors, it can be elicited that the actual and future products proposed for extended delivery of drugs to the posterior portion of the eye, which will surpass the transscleral barrier, must have such features as gradual drug release and a parallel diminished clearance by the organism blood and lymph vessels to be potential candidates for patented formulations [170].

#### 2.3.3. Nanosuspensions

These formulations are obtained using surfactants or polymers to stabilize the particles constituted by drugs, but on a submicron range which, by turn, form colloidal dispersions. This system is quite appropriate to encapsulate hydrophobic drugs. Applying such formulation to the ocular route of delivery yields optimal outcomes when compared to other usual ophthalmic products—diminished irritation, better profile as eye drops, augmented half-life time in the precorneal tissue, sterilization of the product, and improved capacity to solubilize hydrophobic drugs in the lacrimal fluid [171,172]. Some of these effects were also tested in the improved solubilization of glucocorticoids using nanosuspensions for ocular purposes. When referring to pathological conditions in which a variable degree of inflammation is present, disturbing the anterior portion of the ocular tissues, the glucocorticoids as dexamethasone, hydrocortisone, and prednisone are the preferential drugs to treat such illnesses. Nowadays, a therapeutic scheme uses recurrent high doses of the previous pharmacological agents with further initiation of cataract, optic nerve damage, and even glaucoma as undesirable side effects. On behalf of this, the glucocorticoids were so formulated with the model of nanosuspensions to increase the ocular bioavailability of the mentioned drugs [173,174]. Studies conducted by Kassem et al. compared two variables: The ocular bioavailability of glucocorticoid molecules as formulated in microcrystalline suspensions, nanosuspensions, and solutions. The intraocular pressure on the rabbit eye was assessed, for a period up to 12 h, after instillation of the given formulations in the previous referred structure. The results showed that all suspensions presented higher area under the curve (AUC) values in comparison to solutions of drugs with the drugs formulated in nanosuspensions determined to have extensive absorption and increased therapeutic effect in relation to drug solutions [171,175]. Other research carried out by Ali and collaborators related the variable bioavailability of hydrocortisone nanosuspensions formulated using precipitation methodologies with the same parameter on hydrocortisone solutions settled with milling apparatus. The research group used as a rabbit as a model and the formulations were applied as a topical instillation. Here, again, nanosuspensions revealed better results with significant increased bioavailability in comparison to solutions [176]. Using these studies, it can be logically inferred that nanosuspensions can be outlined as efficient formulations to optically deliver poor soluble drugs and can even be merged with hydrogels or ocular implants.

### 2.4. Microneedles Technology

The technology based on the development and production of microneedles is based on a preference for minimal invasiveness. These microneedles possess the capacity to deliver a vast array of drugs to ocular tissues located in the posterior portion of the eye. Along with this, some aspects of the therapeutics may be overcome as the case of the intravitreal injections which possess many frightful consequences such as cataracts, endophthalmitis, hemorrhage, and retinal detachment, allowing to surpass the previous implications. The therapeutic outcomes may also be increased in such diseases as diabetic retinopathy and age-related macular degeneration [149,150,151,152]. Also, this strategy has the capacity to exceed the blood–retinal barrier and deliver higher quantities of drug to the inner portions of the eye preventing also the distribution to unwanted tissues. The formulation of microneedles is also customized to create structures which will only penetrate hundreds of micrometers in the sclera of the eye to circumvent possible damages to deeper assemblies in the eye. With the employment of microneedles, it is possible to specifically place the drug or drug systems into the eye sclera or other intermediate space, namely the suprachoroidal space [153,154,155]. A study using eyes of human corpses demonstrated that microneedles coated with drugs, namely sulforhodamine, verified rapid dissolution of the therapeutic molecule within the sclera [156]. Another study proved that microneedles wrapped with drugs formulated as nanoparticles or microparticles were able to deliver to sclera an infusion of, approximately, 10 to 35 µL of drug solution, confirming the enormous potential of microneedles to be employed in ocular delivery of drugs with few invasiveness [158]. Another study carried out by Patel et al. was able to demonstrate that microneedles optimized for suprachoroidal delivery were safe enough to be applied obtaining also suitable patterns of controlled drug release [159].

## 3. Ocular Gene Delivery

In the last three decades until now gene therapy has achieved a tremendous increase in acceptance from academic milieu and from clinical centers around the world. This technique hires viral vectors which carry material with genetic nature to be specifically delivered into the desired cells. Despite this, the overall process is not perfect and systemic toxicity effects arose. Further developments in this field lead to new avirulent viral strategies with improved capacity to target a tissue (Table 4). The herpes simplex virus-1 ribonucleotide reductase-deficient (RR(-)HSV1) is considered one of the best systems to be pragmatic in the vectorization process of genomic material [177]; inclusively, the United States have a declared patent recognizing the attempt to incorporate a bovine gene for bFGF in the RR(-)HSV1 with posterior analysis in animals revealing fruitful incorporation [178]. This and other backgrounds captured the attention of several researchers for the possibility to use these concepts and deliver other molecules with significant biological roles as cytokines, neurotrophins, or growth factors to be applied in other body tissues [179,180,181].

In another study, the segment of genomic DNA which encodes the information for pigment epithelium-derived factor (PEDF), a proved molecule in the process of protection contrary to neovascularization of choroid membrane, was delivered using a recombinant adeno-associated viral vector (rAAV) to increase the expression of the related gene, with the obvious phenotypic consequences. The researchers used the mouse animal model and the results showed a successful delivery of the information of interest regarded as diminished sizes of the choroid neovascularization lacerations, induced by laser application, observed after injection of the formulation. To properly achieve results the delivered material must contain tissue-specific promoters, several enhancers and other factors for efficient transcription and translation of the gene(s) of interest [182]. 

Another technique relies on the use of short-interfering RNA (siRNA) which can modulate the expression of a given gene according to the correspondence between the sequence of the siRNA molecule and the endogenous mRNA to be silenced. The exogenous molecule is easily delivered through the cell membrane and, in virtue of such, a fresh patent declared the formulation of a cationic polymer which was synthesized and further complexed with the molecule of siRNA. Using the ratio between the number of genomic molecules and cations present, the overall electrical charge of the formulation is possible to be modulated—positive, negative or neutral charge. In a study carried out the siRNA molecules were pointed as inhibitors for mRNA responsible for angiogenic processes, related with the receptors for VEGF1, VEGF2, and VEGF3. The results were clear enough to demonstrate the capacity of the administered formulation to diminish the expression of the gene of interest by 20% [196].

Chalberg et al. filled out a patent process related with the delivery of nucleic acids to the ocular globe. The methodology is based on the capacity of a high-intensity current to permeate the cell membrane generating a mechanical stress in the cell apparatus [197]. After this, the genomic information encoded by a nucleic acid molecule enters the cell and is translated into therapeutic protein molecules. This type of procedure may be used for treatment of such diseases as glaucoma or age-related macular degeneration. Further, the reported gene was used to spot the transfected conjunctival tissues but without using the previously-mentioned patented technique and then compared with the results of the used method. The analysis of the outcomes demonstrated a bioluminescence 2-fold higher in the tissues transfected with the patented methodology in comparison to the procedure without this technique, revealing a successful process of transfection in the first case [198]. Currently, quite a range of researchers worldwide are involved in the development of new viral vectors to avoid toxic effects after the administration via ocular route and to circumvent other reactions, such as tumor development and multiple organ failure. Although the experiences in animal models have been a success, a much more careful planning and regulation of these complex, dangerous, but, in parallel, extremely advantageous systems is required, to be applied in human trials to further assess possible extrapolations targeting future therapeutics for until now uncurable diseases [199,200].

## 4. Conclusions

For quite a few decades, the mechanism of specific drug delivery to ocular milieu was indeed a challenge [201,202]. The common pharmaceutical formulations applied to this purpose, drugs applied as eye drops, suffered downsides which, by turn, required the implementation of distinct carriers aimed in ocular administration route. Aiming to surpass such limitations, deep investments have been made in the field of ocular research to produce safe systems allowing higher rates of therapeutic efficacy and better compliance by patients. Nowadays, scientists are devoting their efforts to achieve advances on in vivo models to further apply the upcoming innovations in clinicals trials. Besides, the dawn of the nanotechnological era with forthcoming production methods and arising technologies with supplementary possible applications on ocular delivery systems developed a growing interest in the scientific community working on this field [203,204,205]. New methodologies for drug encapsulation are being carried out to engineer these nanosystems which will then be administered according to invasive, non-invasive or slightly invasive techniques. In parallel with these advances, the fresh systems—such as dendrimers, liposomes, nanomicelles, nanostructured lipid carriers (NLC), and solid lipid nanoparticles (SLN)—are being thoroughly studied but with few commercially-available options. The preclinical safety of lipid nanoparticles (SLN, NLC) has already been reported for a range of applications including its use as ocular drug delivery systems [206]. The development of these nanotechnological approaches brings new possibilities to therapeutics by reducing the side effects frequently induced by intrinsic toxicity of the drug molecules on the organism of the patient which can inclusively lead to loss of vision. Additionally, the innovative technologies possess other advantages to such therapeutic outcomes as the capacity to be more specific to certain tissues using specific superficial molecules supporting the reduction of the administered dose frequency and even the pharmacokinetic profile of the medicine. Nevertheless, the necessity for vectorized systems to the ocular tissue is still a requirement, especially for tissues localized in the posterior portion of the eye avoiding also an invasive procedure. Thus, in the near future, the delivery systems to be commercialized will certainly exceed current invasive techniques.

## Figures and Tables

**Table 1:** Types of ocular drug delivery systems.

Topical dosage forms	Conjunctival Inserts
	Contact Lenses
	Gels
	Nanoparticles
	Mucoadhesive Polymers
	Ointments
	Solutions
	Suspensions
Intraocular dosage forms	Implants
	Nanoparticles
	Inserts

**Table 2:** Therapeutic molecules, biological targets and potential clinical applications.

Patented System	Clinical Applications	Year	References
Derivatives of Omega chain, hydroxyeicosatetraenoic acid, and 15-hydroxyeicosatetraenoic acid	Dry eye	2001, 2003	[17,18]
Isolated de novo polypeptides	Combination of inhibition of proliferation of HUVEC cells with anti-VEGF activity	2004	[19]
Analogue peptides to bind the sequences Pro-Gly-Pro chemotactic factors of polymorphonuclear leukocytes	Several ocular illnesses and ocular injuries induced by alkali compounds	2004	[20]
Piperidinyl prostaglandin E derivatives	Glaucoma	2005	[21]
Agonist of EP-4	Glaucoma	2004	[22]
Flurbiprofen and ketorolac amide-derivatives for topical administration	Inflammation and angiogenesis of the ocular mucosa	2002	[23]
Solution of concentrated trypsin	Cataracts in the eye	1978	[24]
Toxin extracted from *Clostridium* spp.	Uveitis or inflammation of the ocular mucosa	2004	[25]
Lipopolysaccharides aimed to augment the levels of polypeptide beta defensine-2 (hBD-2)	Infections and wounds in cornea	2006	[26]
Calcium blockers	Proliferative Vitreoretinopathy	2003	[27]
Analogues of prostaglandins	Glaucoma	2008	[28]
Derivatives of dithiolane to inhibit peroxisome proliferator-activated receptor-alpha and gamma	Illnesses of posterior eye portion	2000	[29]
Derivatives of indazole to inhibit tyrosine kinase	Illnesses of posterior eye portion	2006	[30]

**Table 3:** Drug delivery systems for ocular applications.

Patented System	Clinical Applications	Year	Reference
Topical delivery systems containing neurotransmitters and neuropeptides	Dry eye	2008	[76]
Topical delivery system containing modulators of the adhesion molecule for neural cells—polysialic acid—as INF, EGF, and NGF	Dry eye	2008	[77]
Several formulations as in situ gels, solutions and suspensions for delivery of BOL-303213-X	Anti-angiogenic and anti-inflammatory effects in the eye	2008	[78]
Hydrophilic gel for delivery of lidocaine hydrochloride	Local anesthesia of the eye	2008	[79]
Intravitreal injection of anti-VEGF molecule using both lyophilized and unchanging liquid forms	Handling of illnesses related with the posterior segment of the eye	2007	[80]
System to deliver a drug to cornea and sclera stromal cells with a hollow microneedle	Handling of illnesses related with the anterior and posterior segments of the eye	2007	[81]
Encapsulated small and macromolecules for suprachoroidal delivery	Handling of illnesses related with the posterior segment of the eye	2007	[82]
Metal chelator, EDTA, and transport enhancer, methylsulfonylmethane, as eye drop formulation	Diabetic conditions	2010	[83]
Rapamycin as self-emulsion	Handling of illnesses related with the posterior segment of the eye	2006	[84]
Microparticles-engineered formulation containing rapamycin	Wet Age-related Macular Degeneration	2005	[85]
Tacrolimus delivered as formulated eye drops or ocular ointment	Immune-related illnesses of the anterior section of the eye	2003	[86]
Oral formulation containing essential fatty acids omega-3 and omega-6	Dry eye	2006	[87]
A formulation containing amino acids, minerals, phytonutrients and vitamins to nurture the eye	Handling of illnesses related with the anterior and posterior segments of the eye	2003	[88]
A formulation for topical application containing ketotifen fumarate	Antiallergenic	2005	[89]
A jelly formulation with pilocarpine hydrochloride was engineered through combination of Carbopol and Pluronic	Glaucoma	2003	[90]

**Table 4:** Ocular delivery systems for gene therapy.

Patented System	Clinical Application	Year	Reference
Methodology for gene detection with a parallel development of an animal model to assess the effectiveness of the developed compound against the target genes	Genetic pathologies implicated in age-related Macular Degeneration (AMD) and destruction of Retinal Pigment Epithelia	2009	[183]
Lentiviral vectors aiming the transduction of inactive and mitotically active cells	Proliferative ocular disease	2006	[184]
Delivery system for genes as recombinant vectors	Retinal illnesses associated with lysosomal disorders of storage	2003	[185]
Adeno-associated recombinant viral vector for delivery of antiangiogenic factors	Handling of illnesses related with the posterior segment of the eye	2002	[186]
Diagnostic test to identify aberrant pitx3 polypeptides	Dysgenesis of the mesenchymal anterior portion or cataracts	2001	[187]
Inhibitor of RTP801 gene	Microvascular illnesses of the eye	2011	[188]
GFR alpha3 agonist	Retinal disorders	2008	[189]
Modulation of gene expression through narrow band multichromatic electromagnetic radiation	Ocular disorders	2010	[190]
Formulation with 15-lipoxygenase-1 gene to substitute epithelium from the surface of the eye	Dry eye in postmenopausal women	2006	[191]
Method for identification of freac3 gene	Susceptibility of humans to glaucoma and anterior segment dysgenesis	2003	[192]
Hyaluronic acid dihydrazide-derivated or conjugated with nucleic acids	Dry eye syndrome	2003	[193]
Viral vector system for delivery system of glial cell-derived neurotrophic factor	Ocular illnesses	2003	[194]
Novel electroporation methodology to carry RNA molecules to specific spots in the eye	Ocular gene-controlled expression	2005	[195]

## References

[1] Willoughby C.E., Ponzin D., Ferrari S., Lobo A., Landau K., Omidi Y. (2010). Anatomy and physiology of the human eye: Effects of mucopolysaccharidoses disease on structure and function—A review. Clin. Exp. Ophthalmol..

[2] Cholkar K., Dasari S.R., Pal D., Mitra A.K., Mitra A.K. (2013). 1—Eye: Anatomy, physiology and barriers to drug delivery. Ocular Transporters and Receptors.

[3] Gaudana R., Ananthula H.K., Parenky A., Mitra A.K. (2010). Ocular Drug Delivery. AAPS J..

[4] Nagai N. (2016). Design of Novel Ophthalmic Formulation Containing Drug Nanoparticles and Its Usefulness as Anti-glaucoma Drugs. Yakugaku Zasshi.

[5] Tiwari R., Pandey V., Asati S., Soni V., Jain D. (2018). Therapeutic challenges in ocular delivery of lipid based emulsion. Egypt. J. Basic Appl. Sci..

[6] Janagam D.R., Wu L., Lowe T.L. (2017). Nanoparticles for drug delivery to the anterior segment of the eye. Adv. Drug Deliv. Rev..

[7] Sánchez-López E., Espina M., Doktorovova S., Souto E.B., García M.L. (2017). Lipid nanoparticles (SLN, NLC): Overcoming the anatomical and physiological barriers of the eye—Part I - Barriers and determining factors in ocular delivery. Eur. J. Pharm. Biopharm..

[8] Moisseiev E., Loewenstein A. (2017). Drug Delivery to the Posterior Segment of the Eye. Dev. Ophthalmol..

[9] Janoria K.G., Hariharan S., Dasari C.R., Mitra A.K. (2007). Recent patents and advances in ophthalmic drug delivery. Recent Pat. Drug Deliv. Formul..

[10] Tatham A.J., Sarodia U., Gatrad F., Awan A. (2013). Eye drop instillation technique in patients with glaucoma. Eye.

[11] Kati-Sisko V., Hellinen L. (2018). Expression, activity and pharmacokinetic impact of ocular transporters. Adv. Drug Deliv. Rev..

[12] Sarraf D., Lee D.A. (1994). The role of iontophoresis in ocular drug delivery. J. Ocul. Pharm..

[13] Soliman O.A.E., Mohamed E.A.M., El-Dahan M.S., Khatera N.A.A. (2017). Potential Use of Cyclodextrin Complexes for Enhanced Stability, Anti-inflammatory Efficacy, and Ocular Bioavailability of Loteprednol Etabonate. AAPS Pharm. Sci. Tech..

[14] Abdelkader H., Fathalla Z., Moharram H., Ali T.F.S., Pierscionek B. (2018). Cyclodextrin Enhances Corneal Tolerability and Reduces Ocular Toxicity Caused by Diclofenac. Oxid. Med. Cell. Longev..

[15] Kuno N., Fujii S. (2011). Recent Advances in Ocular Drug Delivery Systems. Polymers.

[16] Lavik E., Kuehn M.H., Kwon Y.H. (2011). Novel drug delivery systems for glaucoma. Eye.

[17] Belanger D. (2001). Omega Chain Modified 15-Hydroxyeicosatetraenoic Acid Derivatives and Methods of Their Use for the Treatment of Dry Eye. U.S. Patent.

[18] Klimko P.H., Hellberg M.R., Falck J.R., Conrow R.E. (2003). Hydroxyeicosatetraenoic Acid Analogs and Methods of Their Use in Treating Dry Eye Disorders. U.S. Patent.

[19] Klagsbrun M.S., Soker S. (2004). Peptide Antagonists of Vascularendothelial Growth Factor. U.S. Patent.

[20] Haddox J.P., Pfister R.R., Blalock J.E., Villain M. (2004). Synthetic Complementary Peptides and Ophthalmologic Uses Thereof. U.S. Patent.

[21] Old D.D., Dinh D.T. (2005). Piperidinyl Prostaglandin e Analogs. U.S. Patent.

[22] Woodward D.K., Krauss A.H., Burk R.M., Holoboski M., Posner M.F. (2005). EP4 Agonists as Agents for Lowering Intraocular Pressure. U.S. Patent.

[23] Graff G.H., Hellberg M.R., Yanni J.M. (2003). Method of Treating Ocular Inflammatory and Angiogenesis-Related Disorders of the Posterior Segment of the Eye Using an Amide Derivative of Flurbiprofen or Ketorolac. U.S. Patent.

[24] Spina J.W., Weibel M.K. (1986). Intralenticular Cataract Surgery. U.S. Patent.

[25] First E. (2007). Methods and Compositions for Treating Eye Disorders. U.S. Patent.

[26] Fleiszig S.M., McNamara N.A. (2006). Use of Lipopolysaccharides to Manage Corneal Infections and Wounds. U.S. Patent.

[27] Dreyer E. (2003). Calcium Blockers to Treat Proliferative Vitreoretinopathy. U.S. Patent.

[28] Dinh D. (2008). Prostaglandin Analogs. U.S. Patent.

[29] Pershadsingh H. (2000). 1,2-Dithiolane Derivatives. U.S. Patent.

[30] Borchardt A.J., Kania R.S., Palmer C.L. (2006). Indazole Compounds and Pharmaceutical Compositions for Inhibiting Protein Kinases, and Methods for Their Use. U.S. Patent.

[31] Coroi M.C., Bungau S., Tit M. (2015). Preservatives from the eye drops and the ocular surface. Romanian J. Ophthalmol..

[32] Saettone M.F., Chetoni P., Cerbai R., Mazzanti G., Braghiroli L. (1996). Evaluation of ocular permeation enhancers: In vitro effects on corneal transport of four β-blockers, and in vitro/in vivo toxic activity. Int. J. Pharm..

[33] Furrer P., Mayer J.M., Plazonnet B., Gurny R. (2002). Ocular tolerance of absorption enhancers in ophthalmic preparations. AAPS Pharm. Sci..

[34] Kompella U.B., Kadam R.S., Lee V.H.L. (2010). Recent advances in ophthalmic drug delivery. Ther. Deliv..

[35] Morrison P.W., Khutoryanskiy V.V. (2014). Advances in ophthalmic drug delivery. Ther. Deliv..

[36] Hornof M.D., Bernkop-Schnurch A. (2002). In vitro evaluation of the permeation enhancing effect of polycarbophil-cysteine conjugates on the cornea of rabbits. J. Pharm. Sci..

[37] Kesavan K., Kant S., Singh P.N., Pandit J.K. (2011). Effect of hydroxypropyl-beta-cyclodextrin on the ocular bioavailability of dexamethasone from a pH-induced mucoadhesive hydrogel. Curr. Eye Res..

[38] Loftsson T., Stefánsson E. (2002). Cyclodextrins in eye drop formulations: Enhanced topical delivery of corticosteroids to the eye. Acta Ophthalmol. Scand..

[39] Lang J.C. (1995). Ocular drug delivery conventional ocular formulations. Adv. Drug Deliv. Rev..

[40] Mandal A., Pal D., Agrahari V., Trinh H.M., Joseph M., Mitra A.K. (2018). Ocular delivery of proteins and peptides: Challenges and novel formulation approaches. Adv. Drug Deliv. Rev..

[41] Short B.G. (2008). Safety Evaluation of Ocular Drug Delivery Formulations: Techniques and Practical Considerations. Toxicol. Pathol..

[42] Peng C.C., Bengani L.C., Jung H.J., Leclerc J., Gupta C., Chauhan A. (2011). Emulsions and microemulsions for ocular drug delivery. J. Drug Deliv. Sci. Technol..

[43] Tamilvanan S., Benita S. (2004). The potential of lipid emulsion for ocular delivery of lipophilic drugs. Eur. J. Pharm. Biopharm..

[44] Opitz D.L., Harthan J.S. (2012). Review of Azithromycin Ophthalmic 1% Solution (AzaSite(®)) for the Treatment of Ocular Infections. Ophthalmol. Eye Dis..

[45] Ousler G.W., Michaelson C., Christensen M.T. (2007). An evaluation of tear film breakup time extension and ocular protection index scores among three marketed lubricant eye drops. Cornea.

[46] Ursea R., Purcell T.L., Tan B.U., Nalgirkar A., Lovaton M.E., Ehrenhaus M.R., Schanzlin D.J. (2008). The effect of cyclosporine A (Restasis) on recovery of visual acuity following LASIK. J. Refract. Surg..

[47] Dubald M., Bourgeois S., Andrieu V., Fessi H. (2018). Ophthalmic Drug Delivery Systems for Antibiotherapy—A Review. Pharmaceutics.

[48] Lallemand F., Daull P., Benita S., Buggage R., Garrigue J.-S. (2012). Successfully Improving Ocular Drug Delivery Using the Cationic Nanoemulsion, Novasorb. J. Drug Deliv..

[49] Tajika T., Isowaki A., Sakaki H. (2011). Ocular distribution of difluprednate ophthalmic emulsion 0.05% in rabbits. J. Ocul. Pharmacol. Ther..

[50] Liu Y., Lin X., Tang X. (2009). Lipid emulsions as a potential delivery system for ocular use of azithromycin. Drug Dev. Ind. Pharm..

[51] Shen J., Gan L., Zhu C., Zhang X., Dong Y., Jiang M., Zhu J., Gan Y. (2011). Novel NSAIDs ophthalmic formulation: Flurbiprofen axetil emulsion with low irritancy and improved anti-inflammation effect. Int. J. Pharm..

[52] Ambhore N.P., Dandagi P.M., Gadad A.P. (2016). Formulation and comparative evaluation of HPMC and water soluble chitosan-based sparfloxacin nanosuspension for ophthalmic delivery. Drug Deliv. Transl. Res..

[53] Czajkowska-Kosnik A., Sznitowska M. (2009). Solubility of ocular therapeutic agents in self-emulsifying oils. I. Self-emulsifying oils for ocular drug delivery: Solubility of indomethacin, aciclovir and hydrocortisone. Acta Pol. Pharm..

[54] Muchtar S., Almog S., Torracca M.T., Saettone M.F., Benita S. (1992). A submicron emulsion as ocular vehicle for delta-8-tetrahydrocannabinol: Effect on intraocular pressure in rabbits. Ophthalmic Res..

[55] Robin J.S., Ellis P.P. (1978). Ophthalmic ointments. Surv. Ophthalmol..

[56] Scruggs J., Wallace T., Hanna C. (1978). Route of absorption of drug and ointment after application to the eye. Ann. Ophthalmol..

[57] MacKeen D.L. (1980). Aqueous formulations and ointments. Int. Ophthalmol. Clin..

[58] Ditmar M.F., Polin R.A., Ditmar M.F. (2011). CHAPTER 11—Infectious Diseases. Pediatric Secrets.

[59] Ye Z.-K., Li C., Zhai S.-D. (2014). Guidelines for Therapeutic Drug Monitoring of Vancomycin: A Systematic Review. PLoS ONE.

[60] Baranowski P., Karolewicz B., Gajda M., Pluta J. (2014). Ophthalmic Drug Dosage Forms: Characterisation and Research Methods. Sci. World J..

[61] Occhiutto M.L., Freitas F.R., Maranhao R.C., Costa V.P. (2012). Breakdown of the blood-ocular barrier as a strategy for the systemic use of nanosystems. Pharmaceutics.

[62] Fukuda M., Hanazome I., Sasaki K. (2003). The intraocular dynamics of vancomycin hydrochloride ophthalmic ointment (TN-011) in rabbits. J. Infect. Chemother..

[63] Eguchi H., Shiota H., Oguro S., Kasama T. (2009). The inhibitory effect of vancomycin ointment on the manifestation of MRSA keratitis in rabbits. J. Infect. Chemother..

[64] Guilherme V.A., Ribeiro L.N.M., Tofoli G.R., Franz-Montan M., de Paula E., de Jesus M.B. (2017). Current Challenges and Future of Lipid nanoparticles formulations for topical drug application to oral mucosa, skin, and eye. Curr. Pharm. Des..

[65] Batchelor H.K., Marriott J.F. (2015). Formulations for children: Problems and solutions. Br. J. Clin. Pharmacol..

[66] Kalepu S., Nekkanti V. (2015). Insoluble drug delivery strategies: Review of recent advances and business prospects. Acta Pharm. Sin. B.

[67] Yasueda S., Inada K., Matsuhisa K., Terayama H., Ohtori A. (2004). Evaluation of ophthalmic suspensions using surface tension. Eur. J. Pharm. Biopharm..

[68] Edman P. (1994). Pharmaceutical formulations—Suspensions and solutions. J. Aerosol. Med..

[69] Scoper S.V., Kabat A.G., Owen G.R., Stroman D.W., Kabra B.P., Faulkner R., Kulshreshtha A.K., Rusk C., Bell B., Jamison T. (2008). Ocular distribution, bactericidal activity and settling characteristics of TobraDex ST ophthalmic suspension compared with TobraDex ophthalmic suspension. Adv. Ther..

[70] Patel A., Cholkar K., Agrahari V., Mitra A.K. (2013). Ocular drug delivery systems: An overview. World J. Pharmacol..

[71] Farkouh A., Frigo P., Czejka M. (2016). Systemic side effects of eye drops: A pharmacokinetic perspective. Clin. Ophthalmol. (Auckl. N.Z.).

[72] Andrew R., Luecke G., Dozier S., Diven D.G. (2012). A Pilot Study to Investigate the Efficacy of Tobramycin–Dexamethasone Ointment in Promoting Wound Healing. Dermatol. Ther..

[73] Kobashi H., Kamiya K., Shimizu K. (2017). Randomized Comparison Between Rebamipide Ophthalmic Suspension and Diquafosol Ophthalmic Solution for Dry Eye After Penetrating Keratoplasty. J. Ocul. Pharmacol. Ther..

[74] Sultana Y., Maurya D.P., Iqbal Z., Aqil M. (2011). Nanotechnology in ocular delivery: Current and future directions. Drugs Today (Barc).

[75] Liu S., Jones L.W., Gu F.X. (2012). Nanomaterials for Ocular Drug Delivery. Macromol. Biosci..

[76] Ousler G.W., Chapin M.J., Abelson M.B. (2008). Use of Neurotransmitters and Neuropeptides for the Treatment of Dry Eye Diseases and Related Conditions. U.S. Patent.

[77] Gadd M.G., Graff G. (2008). Modulation of Polysialylated Neural Adhesion Molecules (Psa-Ncam) as a Regulator of Ocular Disease. U.S. Patent.

[78] Bartels S.P.L., Lam T.T., Shafiee A., Lin Y.Q. (2008). Delivery System for Antiangiogenic and Antiinflammatory Pharmaceuticals and Method of Use. U.S. Patent.

[79] Alam A., Reichel E., Busbee B. (2008). Aqueous Gel Formulation and Method for Inducing Topical Anesthesia. U.S. Patent.

[80] Furfine E., Dix D., Graham K.S., Frye K. (2009). VEGF Antagonist Formulations Suitable for Intravitreal Administration. U.S. Patent.

[81] Prausnitz M., Jiang N.H., Edelhauser H.F. (2011). Method for Drug Delivery to Ocular Tissue Using Microneedle. U.S. Patent.

[82] Yamamoto R., Conston S., Sierra D. (2007). Apparatus and Formulations for Suprachoroidal Drug Delivery. U.S. Patent.

[83] Bhushan R., Gin J.B. (2010). Prevention and Treatment of Ophthalmic Complications of Diabetes. U.S. Patent.

[84] Dor P., Mudumba S., Nivaggioli T., Weber D.A. (2006). Formulations for Ocular Treatment. U.S. Patent.

[85] Carrasquillo K., Adamis A.P., Miller J.W., Gragoudas E.S. (2005). Drug Delivery Systems and Use Thereof. U.S. Patent.

[86] Chen J.Q., Liu Y. (2008). Pharmaceutical Compositions and Methods for Treating Immune-Response Associated Diseases of the Surface and the Anterior Segment of the Eye. U.S. Patent.

[87] Thornion S., Troyer E. (2006). Treatment for Dry Eye Syndrome. U.S. Patent.

[88] Gorsek W. (2003). Eyesight Enhanced Maintenance Composition. U.S. Patent.

[89] Abelson M., Gomes P.J., Chapin M.J. (2005). Novel Topical Ophthalmic Formulations. U.S. Patent.

[90] Lin H., Sung K.C. (2003). Ophthalmic Drug Delivery Formulations and Method for Preparing the Same. U.S. Patent.

[91] Maulvi F.A., Soni T.G., Shah D.O. (2016). A review on therapeutic contact lenses for ocular drug delivery. Drug Deliv..

[92] Hsu K.H., Gause S., Chauhan A. (2014). Review of ophthalmic drug delivery by contact lenses. J. Drug Deliv. Sci. Technol..

[93] Nasr F.H., Khoee S., Dehghan M.M., Chaleshtori S.S., Shafiee A. (2016). Preparation and Evaluation of Contact Lenses Embedded with Polycaprolactone-Based Nanoparticles for Ocular Drug Delivery. Biomacromolecules.

[94] Kim J., Chauhan A. (2008). Dexamethasone transport and ocular delivery from poly(hydroxyethyl methacrylate) gels. Int. J. Pharm..

[95] Tomar N., Tomar M., Gulati N., Nagaich U. (2012). pHEMA hydrogels: Devices for ocular drug delivery. Int. J. Health Allied Sci..

[96] Klinger D., Landfester K. (2012). Stimuli-responsive microgels for the loading and release of functional compounds: Fundamental concepts and applications. Polymer.

[97] Hiratani H., Fujiwara A., Tamiya Y., Mizutani Y., Alvarez-Lorenzo C. (2005). Ocular release of timolol from molecularly imprinted soft contact lenses. Biomaterials.

[98] Soluri A., Hui A., Jones L. (2012). Delivery of ketotifen fumarate by commercial contact lens materials. Optom. Vis. Sci..

[99] Abbasi E., Aval S.F., Akbarzadeh A., Milani M., Nasrabadi H.T., Joo S.W., Hanifehpour Y., Nejati-Koshki K., Pashaei-Asl R. (2014). Dendrimers: Synthesis, applications, and properties. Nanoscale Res. Lett..

[100] Wu L.P., Ficker M., Christensen J.B., Trohopoulos P.N., Moghimi S.M. (2015). Dendrimers in Medicine: Therapeutic Concepts and Pharmaceutical Challenges. Bioconjugate Chem..

[101] Lee C.C., MacKay J.A., Fréchet J.M.J., Szoka F.C. (2005). Designing dendrimers for biological applications. Nat. Biotechnol..

[102] Vandamme T.F., Brobeck L. (2005). Poly(amidoamine) dendrimers as ophthalmic vehicles for ocular delivery of pilocarpine nitrate and tropicamide. J. Controll. Release.

[103] Yavuz B., Bozdağ Pehlivan S., Ünlü N. (2013). Dendrimeric Systems and Their Applications in Ocular Drug Delivery. Sci. World J..

[104] Beiko G.H.H., Grzybowski A. (2015). Intraocular lens implants: Do they come with a life time guaranty?. Saudi J. Ophthalmol..

[105] Allan B. (2000). Intraocular lens implants: Have come a long way, but the advances are not yet available to all. BMJ.

[106] Tamaddon L., Mostafavi S.A., Karkhane R., Riazi-Esfahani M., Dorkoosh F.A., Rafiee-Tehrani M. (2015). Design and development of intraocular polymeric implant systems for long-term controlled-release of clindamycin phosphate for toxoplasmic retinochoroiditis. Adv. Biomed. Res..

[107] Li X., Kelly D., Nolan J.M., Dennison J.L., Beatty S. (2017). The evidence informing the surgeon’s selection of intraocular lens on the basis of light transmittance properties. Eye.

[108] Lee S.S., Hughes P., Ross A.D., Robinson M.R. (2010). Biodegradable implants for sustained drug release in the eye. Pharm. Res..

[109] Kim Y.C., Chiang B., Wu X., Prausnitz M.R. (2014). Ocular delivery of macromolecules. J. Control. Release.

[110] Hebson C.B., Srivastava S.K. (2011). A functional, nonfunctioning Retisert implant. Ocul. Immunol. Inflamm..

[111] Jaffe G.J., Martin D., Callanan D., Pearson P.A., Levy B., Comstock T. (2006). Fluocinolone Acetonide Implant (Retisert) for Noninfectious Posterior Uveitis. Ophthalmology.

[112] Jancevski M., Foster C.S. (2010). The Retisert Experience. Investig. Ophthalmol. Vis. Sci..

[113] Kuno N., Fujii S. (2010). Biodegradable intraocular therapies for retinal disorders: Progress to date. Drugs Aging.

[114] Ghasemi Falavarjani K. (2009). Implantable Posterior Segment Drug Delivery Devices; Novel Alternatives to Currently Available Treatments. J. Ophthalmic Vis. Res..

[115] Muccioli C., Belfort R. (2000). Treatment of cytomegalovirus retinitis with an intraocular sustained-release ganciclovir implant. Braz. J. Med. Biol. Res..

[116] Dhillon B., Kamal A., Leen C. (1998). Intravitreal sustained-release ganciclovir implantation to control cytomegalovirus retinitis in AIDS. Int. J. STD AIDS.

[117] Mittal S., Miranda O. (2018). Recent Advancements in Biodegradable Ocular Implants. Curr. Drug Deliv..

[118] Sanchez-Lopez E., Egea M.A., Davis B.M., Guo L., Espina M., Silva A.M., Calpena A.C., Souto E.M.B., Ravindran N., Ettcheto M. (2018). Memantine-Loaded PEGylated Biodegradable Nanoparticles for the Treatment of Glaucoma. Small.

[119] Ng X.W., Liu K.L., Veluchamy A.B., Lwin N.C., Wong T.T., Venkatraman S.S. (2015). A biodegradable ocular implant for long-term suppression of intraocular pressure. Drug Deliv. Transl. Res..

[120] Lee D.J. (2015). Intraocular Implants for the Treatment of Autoimmune Uveitis. J. Funct. Biomater..

[121] Haghjou N., Soheilian M., Abdekhodaie M.J. (2011). Sustained Release Intraocular Drug Delivery Devices for Treatment of Uveitis. J. Ophthalmic Vis. Res..

[122] Rishi P., Rishi E., Kuniyal L., Mathur G. (2012). Short-term results of intravitreal dexamethasone implant (Ozurdex^®^) in treatment of recalcitrant diabetic macular edema: A case series. Oman. J. Ophthalmol..

[123] Garweg J.G., Zandi S. (2016). Retinal vein occlusion and the use of a dexamethasone intravitreal implant (Ozurdex^®^) in its treatment. Graefe’s Arch. Clin. Exp. Ophthalmol..

[124] Zucchiatti I., Lattanzio R., Querques G., Querques L., Del Turco C., Cascavilla M.L., Bandello F. (2012). Intravitreal Dexamethasone Implant in Patients with Persistent Diabetic Macular Edema. Ophthalmologica.

[125] Sheshala R., Kok Y.Y., Ng J.M., Thakur R.R., Dua K. (2015). In Situ Gelling Ophthalmic Drug Delivery System: An Overview and Its Applications. Recent Pat. Drug Deliv. Formul..

[126] Kouchak M. (2014). In Situ Gelling Systems for Drug Delivery. Jundishapur J. Nat. Pharm. Prod..

[127] Zahir-Jouzdani F., Wolf J.D., Atyabi F., Bernkop-Schnurch A. (2018). In situ gelling and mucoadhesive polymers: Why do they need each other?. Expert Opin. Drug Deliv..

[128] Van Tomme S.R., Storm G., Hennink W.E. (2008). In situ gelling hydrogels for pharmaceutical and biomedical applications. Int. J. Pharm..

[129] Mundada A.S., Avari J.G. (2009). In Situ Gelling Polymers in Ocular Drug Delivery Systems: A Review. Ther. Drug Carr. Syst..

[130] Cholkar K., Patel S.P., Vadlapudi A.D., Mitra A.K. (2013). Novel Strategies for Anterior Segment Ocular Drug Delivery. J. Ocul. Pharmacol. Ther..

[131] Sanchez-Lopez E., Espina M., Doktorovova S., Souto E.B., Garcia M.L. (2017). Lipid nanoparticles (SLN, NLC): Overcoming the anatomical and physiological barriers of the eye—Part II—Ocular drug-loaded lipid nanoparticles. Eur. J. Pharm. Biopharm..

[132] Gao Y., Sun Y., Ren F., Gao S. (2010). PLGA-PEG-PLGA hydrogel for ocular drug delivery of dexamethasone acetate. Drug Dev. Ind. Pharm..

[133] Rieke E.R., Amaral J., Becerra S.P., Lutz R.J. (2010). Sustained subconjunctival protein delivery using a thermosetting gel delivery system. J. Ocul. Pharmacol. Ther..

[134] Heller J., Schacht E., Toncheva V. (2006). PEG-Polyacetal and PEG-Polyacetal-POE Graft Copolymers and Pharmaceutical Compositions. U.S. Patent.

[135] Akbarzadeh A., Rezaei-Sadabady R., Davaran S., Joo S.W., Zarghami N., Hanifehpour Y., Samiei M., Kouhi M., Nejati-Koshki K. (2013). Liposome: Classification, preparation, and applications. Nanoscale Res. Lett..

[136] Agarwal R., Iezhitsa I., Agarwal P., Abdul Nasir N.A., Razali N., Alyautdin R., Ismail N.M. (2016). Liposomes in topical ophthalmic drug delivery: An update. Drug Deliv..

[137] Mishra G.P., Bagui M., Tamboli V., Mitra A.K. (2011). Recent Applications of Liposomes in Ophthalmic Drug Delivery. J. Drug Deliv..

[138] Natarajan J.V., Ang M., Darwitan A., Chattopadhyay S., Wong T.T., Venkatraman S.S. (2012). Nanomedicine for glaucoma: Liposomes provide sustained release of latanoprost in the eye. Int. J. Nanomed..

[139] Taha E.I., El-Anazi M.H., El-Bagory I.M., Bayomi M.A. (2014). Design of liposomal colloidal systems for ocular delivery of ciprofloxacin. Saudi Pharm. J..

[140] Villasmil-Sánchez S., Drhimeur W., Ospino S.C.S., Rabasco Alvarez A.M., González-Rodríguez M.L. (2010). Positively and negatively charged liposomes as carriers for transdermal delivery of sumatriptan: In vitro characterization. Drug Dev. Ind. Pharm..

[141] Law S.L., Huang K.J., Chiang C.H. (2000). Acyclovir-containing liposomes for potential ocular delivery: Corneal penetration and absorption. J. Control. Release.

[142] Zhang J., Wang S. (2009). Topical use of Coenzyme Q10-loaded liposomes coated with trimethyl chitosan: Tolerance, precorneal retention and anti-cataract effect. Int. J. Pharm..

[143] Habib F.S., Fouad E.A., Abdel-Rhaman M.S., Fathalla D. (2010). Liposomes as an ocular delivery system of fluconazole: In-vitro studies. Acta Ophthalmol..

[144] Dai Y., Zhou R., Liu L., Lu Y., Qi J., Wu W. (2013). Liposomes containing bile salts as novel ocular delivery systems for tacrolimus (FK506): In vitro characterization and improved corneal permeation. Int. J. Nanomed..

[145] Bulbake U., Doppalapudi S., Kommineni N., Khan W. (2017). Liposomal Formulations in Clinical Use: An Updated Review. Pharmaceutics.

[146] Essa L., Laughton D., Wolffsohn J.S. (2018). Can the optimum artificial tear treatment for dry eye disease be predicted from presenting signs and symptoms?. Cont. Lens. Anterior Eye.

[147] Calvao-Santos G., Borges C., Nunes S., Salgado-Borges J., Duarte L. (2011). Efficacy of 3 different artificial tears for the treatment of dry eye in frequent computer users and/or contact lens users. Eur. J. Ophthalmol..

[148] Rosenfeld P.J., Saperstein D.A., Bressler N.M., Reaves T.A., Sickenberg M., Rosa R.H., Sternberg P., Aaberg T.M., Aaberg T.M. (2004). Verteporfin in Ocular Histoplasmosis Study, G. Photodynamic therapy with verteporfin in ocular histoplasmosis: Uncontrolled, open-label 2-year study. Ophthalmology.

[149] Bakri S.J., Kaiser P.K. (2004). Verteporfin ocular photodynamic therapy. Expert. Opin. Pharmacother..

[150] Vadlapudi A.D., Mitra A.K. (2013). Nanomicelles: An emerging platform for drug delivery to the eye. Ther. Deliv..

[151] Trinh H.M., Joseph M., Cholkar K., Mitra R., Mitra A.K., Mitra A.K., Cholkar K., Mandal A. (2017). Nanomicelles in Diagnosis and Drug Delivery (Chapter 3). Emerging Nanotechnologies for Diagnostics, Drug Delivery and Medical Devices.

[152] Cholkar K., Patel A., Vadlapudi A.D., Mitra A.K. (2012). Novel Nanomicellar Formulation Approaches for Anterior and Posterior Segment Ocular Drug Delivery. Recent Pat. Nanomed..

[153] Cholkar K., Gilger B.C., Mitra A.K. (2015). Topical, Aqueous, Clear Cyclosporine Formulation Design for Anterior and Posterior Ocular Delivery. Transl. Vis. Sci. Technol..

[154] Civiale C., Licciardi M., Cavallaro G., Giammona G., Mazzone M.G. (2009). Polyhydroxyethylaspartamide-based micelles for ocular drug delivery. Int. J. Pharm..

[155] Tong Y.C., Chang S.F., Liu C.Y., Kao W.W., Huang C.H., Liaw J. (2007). Eye drop delivery of nano-polymeric micelle formulated genes with cornea-specific promoters. J. Gene Med..

[156] Abhirup Mandal A., Gote V., Pal D., Oguandele A., Mitr A. (2019). Ocular Pharmacokinetics of a Topical Ophthalmic Nanomicellar Solution of Cyclosporine (Cequa^®^) for Dry Eye Disease. Pharm. Res..

[157] Ideta R., Yanagi Y., Tamaki Y., Tasaka F., Harada A., Kataoka K. (2004). Effective accumulation of polyion complex micelle to experimental choroidal neovascularization in rats. FEBS. Lett..

[158] Weng Y.H., Ma X.W., Che J., Li C., Liu J., Chen S.Z., Wang Y.Q., Gan Y.L., Chen H., Hu Z.B. (2018). Nanomicelle-Assisted Targeted Ocular Delivery with Enhanced Antiinflammatory Efficacy In Vivo. Adv. Sci..

[159] Patel S., Berezovsky E., Berezovsky D.E., McCarey B.E., Zarnitsyn V., Edelhauser H.F., Prausnit M.R. (2012). Targeted Administration into the Suprachoroidal Space Using a Microneedle for Drug Delivery to the Posterior Segment of the Eye. Inv. Ophth. Vis. Sci..

[160] Khan I., Saeed K., Khan I. (2017). Nanoparticles: Properties, applications and toxicities. Arabian J. Chem..

[161] Vasconcelos A., Vega E., Pérez Y., Gómara M.J., García M.L., Haro I. (2015). Conjugation of cell-penetrating peptides with poly (lactic-co-glycolic acid)-polyethylene glycol nanoparticles improves ocular drug delivery. Int. J. Nanomed..

[162] Sánchez-López E., Egea M.A., Cano A., Espina M., Calpena A.C., Ettcheto M., Camins A., Souto E.B., Silva A.M., García M.L. (2016). PEGylated PLGA nanospheres optimized by design of experiments for ocular administration of dexibuprofen—In vitro, ex vivo and in vivo characterization. Colloids Surf. B: Biointerfaces.

[163] Al-Halafi A.M. (2014). Nanocarriers of nanotechnology in retinal diseases. Saudi J. Ophthalmol..

[164] Gupta H., Aqil M., Khar R., Ali A., Bhatnagar A., Mittal G. (2013). Nanoparticles laden in situ gel for sustained ocular drug delivery. J. Pharm. Bioallied. Sci..

[165] Ibrahim H.K., El-Leithy I.S., Makky A.A. (2010). Mucoadhesive Nanoparticles as Carrier Systems for Prolonged Ocular Delivery of Gatifloxacin/Prednisolone Bitherapy. Mol. Pharm..

[166] Musumeci T., Bucolo C., Carbone C., Pignatello R., Drago F., Puglisi G. (2013). Polymeric nanoparticles augment the ocular hypotensive effect of melatonin in rabbits. Int. J. Pharm..

[167] Xu Q., Kambhampati S.P., Kannan R.M. (2013). Nanotechnology Approaches for Ocular Drug Delivery. Middle East. Afr. J. Ophthalmol..

[168] Bucolo C., Drago F., Salomone S. (2012). Ocular drug delivery: A clue from nanotechnology. Front. Pharmacol..

[169] Amrite A.C., Edelhauser H.F., Singh S.R., Kompella U.B. (2008). Effect of circulation on the disposition and ocular tissue distribution of 20 nm nanoparticles after periocular administration. Mol. Vis..

[170] Weng Y., Liu J., Jin S., Guo W., Liang X., Hu Z. (2017). Nanotechnology-based strategies for treatment of ocular disease. Acta Pharm. Sin. B.

[171] Kassem M.A., Abdel Rahman A.A., Ghorab M.M., Ahmed M.B., Khalil R.M. (2007). Nanosuspension as an ophthalmic delivery system for certain glucocorticoid drugs. Int. J. Pharm..

[172] Das S., Suresh P.K. (2011). Nanosuspension: A new vehicle for the improvement of the delivery of drugs to the ocular surface. Application to amphotericin B. Nanomed. Nanotechnol. Biol. Med..

[173] Patel V.R., Agrawal Y.K. (2011). Nanosuspension: An approach to enhance solubility of drugs. J. Adv. Pharm. Tech. Res..

[174] Ahire E., Thakkar S., Darshanwad M., Misra M. (2018). Parenteral nanosuspensions: A brief review from solubility enhancement to more novel and specific applications. Acta Pharm. Sin. B.

[175] Soltani S., Zakeri-Milani P., Barzegar-Jalali M., Jelvehgari M. (2016). Comparison of Different Nanosuspensions as Potential Ophthalmic Delivery Systems for Ketotifen Fumarate. Adv. Pharm. Bull..

[176] Ali H.S.M., York P., Ali A.M.A., Blagden N. (2011). Hydrocortisone nanosuspensions for ophthalmic delivery: A comparative study between microfluidic nanoprecipitation and wet milling. J. Control. Release.

[177] Yoon S.S., Carroll N.M., Chiocca E.A., Tanabe K.K. (1998). Cancer gene therapy using a replication-competent herpes simplex virus type 1 vector. Ann. Surg..

[178] Brandt C.R., Kalil R.E., Agarwala S. (2000). Replication Competent, a Virulent Herpes Simplex Virus as a Vector for Neural and Ocular Gene Therapy. U.S. Patent.

[179] Campbell J.P., McFarland T.J., Stout J.T. (2016). Ocular Gene Therapy. Dev. Ophthalmol..

[180] Liu M.M., Tuo J., Chan C.-C. (2011). Republished review: Gene therapy for ocular diseases. Postgrad. Med. J..

[181] Uthra S., Kumaramanickavel G. (2009). Gene therapy in ophthalmology. Oman. J. Ophthalmol..

[182] Hauswirth W., Campichiaro P.A., Berns K.I. (2008). Raav Vector Compositions and Methods for the Treatment of Choroidal Neovascularization. NZ Patent.

[183] Inana G., McLaren M. (2009). Methods and Compositions for Detecting and Treating Retinal Diseases. U.S. Patent.

[184] Stout J.T., Appukuttan B. (2006). Lentiviral Vector-Mediated Gene Transfer and Uses Thereof. U.S. Patent.

[185] Davidson B., Jolly D.J., Sauter S.L., Stein C.S., Dubensky T.W., Heth J.A. (2006). Use of Recombinant Gene Delivery Vectors for Treating or Preventing Lysosomal Storage Disorders. U.S. Patent.

[186] Manning W., Dwarki V.J., Rendahl K., Zhou S., McGee L., Lau D., Flannery J.G., Miller S.S., Wang F., Di Polo A. (2002). Use of Recombinant Gene Delivery Vectors for Treating or Preventing Diseases of the Eye. U.S. Patent.

[187] Murray J.C., Semina E. (2001). Methods and Compositions for the Diagnosis and Treatment of Cataracts. U.S. Patent.

[188] Feinstein E., Skaliter R. (2011). Inhibitors of RTP801 and Their Use in Disease Treatment. U.S. Patent.

[189] Jorgensen J. (2008). Treatment of Retinopathies Using Gfra3 Agonists. U.S. Patent.

[190] McDaniel D. (2006). System and Method for Photodynamic Cell Therapy. U.S. Patent.

[191] Yanni J., Gamache D.A., Miller S.T. (2006). Treatment of Dry Eye Restoring 15-Lipoxygenase Activity to Ocular Surface Cells. U.S. Patent.

[192] Walter M.A., Jordan T., Raymond V. (2003). Novel Mutations in the Freac3 Gene for Diagnosis and Prognosis of Glaucoma and Anterior Segment Dysgenesis. U.S. Patent.

[193] DeHazya P., Chen W. (2003). Gene Therapy for Dry Eye Syndrome. U.S. Patent.

[194] Flannery J., Hauswirth W.W. (2003). Expression of Glial-Derived Neurotrophic Factor for Treatment of Diseases of the Eye. U.S. Patent.

[195] Heller R., Jaroszeski M.J., Gilbert R.A., Hauswirth W.H. (2006). Electroporation Device and Method for Delivery to Ocular Tissue. WO Patent.

[196] Vargeese C., Wang W., Chen T., Sweedler D., Haeberli P. (2003). Polycationic Compositions for Cellular Delivery of Polynucleotides. EP Patent.

[197] Chalberg T.W., Blumenkranz M., Palanker D.V., Vankov A., Huie P., Marmor M.F., Calos M.P. (2007). Ocular Gene Therapy Using Avalanche-Mediated Transfection. U.S. Patent.

[198] McDaniel D.H. (2010). System and Method for Photodynamic Cell Therapy. U.S. Patent.

[199] Petit L., Khanna H., Punzo C. (2016). Advances in Gene Therapy for Diseases of the Eye. Hum. Gene. Ther..

[200] Xue K., Groppe M., Salvetti A.P., MacLaren R.E. (2017). Technique of retinal gene therapy: Delivery of viral vector into the subretinal space. Eye.

[201] Canadas C., Alvarado H., Calpena A.C., Silva A.M., Souto E.B., Garcia M.L., Abrego G. (2016). In vitro, ex vivo and in vivo characterization of PLGA nanoparticles loading pranoprofen for ocular administration. Int. J. Pharm..

[202] Abrego G., Alvarado H., Souto E.B., Guevara B., Bellowa L.H., Parra A., Calpena A., Garcia M.L. (2015). Biopharmaceutical profile of pranoprofen-loaded PLGA nanoparticles containing hydrogels for ocular administration. Eur. J. Pharm. Biopharm..

[203] Fangueiro J.F., Andreani T., Fernandes L., Garcia M.L., Egea M.A., Silva A.M., Souto E.B. (2014). Physicochemical characterization of epigallocatechin gallate lipid nanoparticles (EGCG-LNs) for ocular instillation. Colloids Surf. B Biointerfaces.

[204] Araujo J., Garcia M.L., Mallandrich M., Souto E.B., Calpena A.C. (2012). Release profile and transscleral permeation of triamcinolone acetonide loaded nanostructured lipid carriers (TA-NLC): In vitro and ex vivo studies. Nanomedicine.

[205] Gonzalez-Mira E., Nikolic S., Calpena A.C., Egea M.A., Souto E.B., Garcia M.L. (2012). Improved and safe transcorneal delivery of flurbiprofen by NLC and NLC-based hydrogels. J. Pharm. Sci..

[206] Doktorovova S., Kovacevic A.B., Garcia M.L., Souto E.B. (2016). Preclinical safety of solid lipid nanoparticles and nanostructured lipid carriers: Current evidence from in vitro and in vivo evaluation. Eur. J. Pharm. Biopharm..

